# How firms cope with social crisis: The mediating role of digital transformation as a strategic response to the COVID-19 pandemic

**DOI:** 10.1371/journal.pone.0282854

**Published:** 2023-04-04

**Authors:** Weilin Wu, Huanxiang Wang, Lei Lu, Guangya Ma, Xiaoxiao Gao

**Affiliations:** 1 School of Economics, Jiaxing University, Jiaxing, Zhejiang, China; 2 School of Business, Macau University of Science and Technology, Avenida WaiLong, Macau, China; University of Naples Federico II: Universita degli Studi di Napoli Federico II, ITALY

## Abstract

The COVID-19 pandemic has drawn attention to the strategic responses of Chinese firms on digital transformation and led to a call for enhancing competitive advantage via accelerating digital transformation. Besides the physical health issue, the pandemic has triggered an extraordinary social and economic crisis in which service industries have been attacked hard. In this situation, firms are meeting increasing competitive pressure, which urges them to achieve better performance with the help of digital transformation. Based on the technology-organization-environment framework and dynamic capabilities theory, this research proposed two studies with two methods, including a structural equation model and a regression discontinuity design with a fixed-effect model. The findings suggest digital transformation mediates the relationship between competitive pressure and firm performance among Chinese small- and medium-sized enterprises and large firms after the outbreak of COVID-19, respectively. It confirms that digital transformation is a practical strategic decision for Chinese service firms to respond to increasing competitive pressure in the COVID-19 pandemic. Besides, the results also illustrate the moderating effects of absorptive, innovative, and adaptive capability on the relationship between digital transformation and firm performance among large firms.

## 1. Introduction

After the outbreak of COVID-19 at the end of 2019, corporate operations and personal lives have been hit hard by the unprecedented global pandemic, and the service industry is reported to be one of the industries most affected by the COVID-19 pandemic [1]. The service industry requires more professionals to provide services directly, such as consulting, tourism, and education, instead of machines. In the background of the pandemic, some service companies that offer essential services (e.g., logistics, retailing, and health care) were required to continue to serve in order to maintain the city running, but they need to improve their working model to protect employees and customers. Secondly, due to the necessity of pandemic prevention, some healthy measures were required in corporate operations and daily activities, which challenged businesses’ development. For example, because of the lockdown policy, some service providers like hairdressers, hotels and airlines failed to operate [2]; because of the physical distance and quarantine measure, some service providers from the fields of consulting, media and education were unable to engage in offline activities and had to develop their online service business [1]. They have to seek new ways of connecting with customers and offering their services. For instance, exploiting online office and cloud meetings can avoid physical connections; online orders and sales can decrease firms’ fixed costs. Digital transformation provides these service providers an alternative strategic choice to cope with this social crisis. Also, with the growing scale of the service industry in the national economy, helping enterprises in the service industry cope with a social crisis can reduce the unemployment rate and maintain social stability. Thus, we attempt to determine whether digital transformation can help service enterprises cope with the challenges of the COVID-19 pandemic [3].

On the one hand, some believe it is currently an uncertain and complex business environment in which firms face severe challenges [4]. On the other hand, some argue that proper strategic responses enable firms actively adapt to the volatile environment and perform better [5]. Among many alternative strategies, digital transformation is considered one of the most beneficial business competitive strategies, because it can help firms keep a continuous competitive in a rapidly changing business context [6]. Although it has been reported that the COVID-19 pandemic has accelerated firms’ digital transformation [4], it has yet to explore widely whether digital transformation is a practical strategic response to the outbreak of COVID-19 for Chinese firms from the service sector and whether digital transformation can improve firm performance under an environment with more substantial competitive pressure like the environment in the COVID-19 pandemic.

This study considers digital transformation a strategic decision that responds to the dynamic environmental change triggered by the COVID-19 pandemic. Some necessary prevention measures in the pandemic, like No dine-in and home quarantine, decrease business opportunities and exacerbate the competitive pressure among enterprises. According to the technology-organization-environment (TOE) framework, competitive pressure in an environmental context is one of the significant antecedents to promote firms’ transformation or adoption behavior of innovative technology [7]. However, the influence of increasing competitive pressure from an environmental perspective on firms’ strategy has yet to be considered in the COVID-19 pandemic.

Besides, the coming issue is how firms exploited digital transformation to improve their performance, especially during the COVID-19 pandemic. Some reported that firms’ digital transformation hardly brought better performance, but brought economic loss and technological failures [8]. Thus, it suggests that the Critical for effective digital transformation is the capability to know the usage of technical applications [9]. In this research, we believe firms need digital transformation to improve their performance. Also, they need to have the ability to adopt new technology during the process when digital transformation influences firm performance. After coordinating new knowledge and new resources, digital transformation can be integrated better into firms’ operations, routine activities and core tasks [10]. Therefore, based on the dynamic capabilities theory, these capabilities might be boundary conditions that would reinforce the positive effect of digital transformation on firm performance. Prior studies mainly focused on the relationships between dynamic capabilities, organizational behavior, and organizational outcome [11,12] and the moderating role of a single dimension in dynamic capabilities in the process of corporate governance [13–15], but they failed to consider the moderating roles of multiple dimensions in dynamic capabilities, including absorptive capability, adaptive capability and innovative capability [16], in the process of digital transformation. Therefore, we also test the possibility of dynamic capabilities influencing the effect of digital transformation on firm performance in a strong competitive environment like the COVID-19 pandemic.

Furthermore, past studies mentioned the differences between small- and medium-sized enterprises (SMEs) and large enterprises when they dealt with public crises and introduced new technology [11,17]. Some suggested that SMEs are more vulnerable to uncertain environments due to limited resources [18]. And, it also was argued that large enterprises need more time executing transformation, because their established operation frameworks in professional fields are too reliable to change [9]. Thus, this research views firms from the service sector in the light of competitive pressure, digital transformation and firm performance in the COVID-19 pandemic, in order to examine the details of SMEs compared to large enterprises. And, we also explore the role of dynamic capabilities in the digital transformation process among large companies. To achieve the research objective, we propose the following research questions (RQ):

RQ1: Does competitive pressure promote firms’ digital transformation in the COVID-19 pandemic? Does firms’ digital transformation improve their performance?

RQ2: Is digital transformation a beneficial strategic response to the COVID-19 pandemic for SMEs and large enterprises? And what effects do dynamic capabilities have on digital transformation and firm performance among large companies?

To answer these questions, this study conducted two studies with the theoretical help of competitive pressure from the TOE framework [19], and absorptive capability, innovative capability and adaptive capability from dynamic capabilities theory [20]. The first study exploited a set of cross-sectional survey data from Chinese SEMs’ senior managers or owners who have been involved in digital transformation in the service sector collected in 2022. The second study exploited longitudinal data on a sample of Chinese public firms in the service sector from 2018 to 2020. With the help of the structural equation model (SEM) and fixed-effect model, this study attempted to find whether digital transformation is a proper strategic choice for SMEs and large enterprises to respond to increasing competitive pressure after the outbreak of COVID-19.

This study makes several contributions. First, prior research confirmed the effect of competitive pressure on digital transformation in the manufacturing industry [21]. Still, they have yet to profoundly examine the impact of competitive pressure on digital transformation in the service industry. Under the TOE framework, we identify competitive pressure as the antecedent of digital transformation in Chinese SMEs and large firms from the service industry. Second, focusing on digital transformation in firms from the sector industry, this research contributes to the literature and tests the significant achievement of digital transformation in the service sector as a strategic response to the COVID-19 outbreak. Third, combined with dynamic capabilities theory, this study explores the boundary condition in which firms’ digital transformation under the TOE framework provides a theoretical supplement for understanding the TOE framework. Lastly, this research enriches the comparative investigation of SEMs and large enterprises’ digital transformation under a social crisis via two separate study designs with different contexts and data collection approaches. Given our two studies with two methodologies, our reports achieve stronger generalizability and robustness.

## 2. Previous research and hypothesis development

### 2.1 TOE framework and competitive pressure

The TOE framework is considered as a significant theoretical perspective for exploring contextual determinants of firms’ strategic innovative decisions [19]. The TOE framework declares three factors that affect organizational adoption towards technological innovation, including the technological context, the organizational context and the environmental context [22]. The technological context is related to technology and abilities which are available in the organization [19]. The organizational context refers to internal criteria, such as organizational size, sales, etc [22]. The environmental context emphasizes the external field where an organization operates, including related industries and competitors [19]. The TOE framework has been applied widely to assess firms’ adoption behavior towards innovative technology, e.g., green information technology [23], customer relationship management [7], electronic data interchange [24], cloud computing [25], e-business [19], etc. Table 1 lists studies about firms’ innovative behaviors under the TOE framework or competitive pressure.

**Table 1:** Research on the TOE framework and competitive pressure.

Study	Research area	Constructs	Findings
Chen et al., 2022 [26]	The factors influencing performance of using artificial intelligence in hospitality industry	System quality, perceived risk, management support, innovativeness, competitive pressure, regulatory support	The analysis results reveal that perceived AI risk, management support, innovativeness, competitive pressure and regulatory support significantly influence the performance of AI adoption
Bag et al., 2022 [27]	The antecedents of blockchain technology adoption and its effect on SMEs’ performance	Relative advantage, compatibility, complexity, top management support, organizational readiness, competitive pressure, external support from vendors, regulation and legislations, blockchain adoption, financial performance, market performance	Relative advantage, compatibility, top management support, organizational readiness, competitive pressures, external support, regulations and legislation significantly impact SMEs’ blockchain adoption
Salah et al., 2021 [28]	The using of Customer Relationship Management in the Palestinian SMEs	Compatibility, IT infrastructure, competitive, relative advantage, security, Top management, competitive pressure, customer pressure, firm size, Customer Relationship Management adoption	Firm size moderates the relationship between top management support, compatibility, customer pressure, IT infrastructure, and Customer Relationship Management adoption.
Cruz-Jesus et al., 2019 [7]	The antecedents that affect Customer Relationship Management adoption stages in firms	Technology competence, data quality and integration, top management support, competitive pressure, CRM evaluation, CRM adoption, CRM routinization	The intention to adopt CRM in firms is predicted jointly and positively by technology competence, data quality and integration, top management support and CRM evaluation, but competitive pressure negatively influence CRM adoption
Zhu, Dong, et al., 2006 [19]	The determinants of E-business usage in post-adoption stages of innovation diffusion among European firms	Relative advantage, compatibility, costs, security concern, technology competence, organization size, competitive pressure, partner readiness, E-business usage, E-business impact	Compatibility, relative advantage, technology competence, partner readiness and competitive pressure significantly drive e-business usage. Security concern, cost, and organization size significantly inhibit e-business usage.
Oliveira et al., 2014 [25]	The adoption of cloud computing in the manufacturing and services sectors	Security concerns, cost savings, relative advantage, complexity, compatibility, cloud computing adoption, technology readiness, top management support, firm size, competitive pressure, regulatory support	The relative advantage is positively influenced by cost savings. The adoption is determined by relative advantage, complexity, technological readiness, top management support, and firm size.
Ramakrishnan et al., 2012 [29]	The determinants of influencing business intelligence (BI) data collection strategies	Institutional isomorphism, competitive pressure, insight, consistency, organizational transformation, problem driven strategy for BI data collection, comprehensive data collection strategy for BI data collection	The implementing BI for the purpose of achieving consistency is predicted by institutional isomorphism, and comprehensive data collection strategy for BI data collection is predicted jointly by consistency and organizational transformation.
Thong, 1999 [30]	The determinants of using Information System (IS) in small businesses	CEO’s innovativeness, CEO’s IS knowledge, relative advantage of IS, compatibility of IS, complexity of IS, business size, employees’ IS knowledge, information intensity, competition.	CEO’s innovativeness and IS knowledge, relative advantage, compatibility, complexity of IS, business size and level of employees’ IS knowledge collectively influence the likelihood of adoption of IS in small businesses.

Past studies on digital transformation and competitive pressure received the theoretical support of the TOE framework [7,25,31]. Thus, the TOE framework is a widely accepted theoretical framework investigating the influence of competitive pressure on digital transformation. In this research, we focus on competitive pressure from the environmental context in the TOE framework. Competitive pressure refers to the level of pressure perceived by an organization from its competitors in a market [31,32]. Previous studies confirmed the significance of competitive pressure when an organization adopts innovative technology, as shown in Table 1. Furthermore, firms’ business environment has been plagued by uncertainty during the COVID-19 pandemic, which brings more challenges to the survival and development of firms. The rapid change in the business environment makes the competition between firms more intense, because some pandemic prevention policies, such as keeping distance and higher health standards, reduce business opportunities and increase business costs. In this background, firms’ business environment has undergone a noticeable change. Thus, competition pressure from the environmental context is considered an essential driver of firms’ transformation to respond to the new challenges of the COVID-19 pandemic.

Competitive pressure makes firms remain vigilant to avoid failure in the industry competition. Fierce competitive pressure forces firms to make change and occupy more market shares [33]. These firms which prefer to adopt innovative technology enhances the flexibility and stability of operations, and integrates differentiation into firms’ services and products [34], which updates the industry standards, elevates industry barriers and establishes new industry benchmark of being the leader [21]. So, the hypothesis is proposed:

**Hypothesis 1.** *Competitive pressure is positively related to firms’ performance during the COVID-19 pandemic*.

### 2.2 Digital transformation and competitive pressure

Firms’ digital transformation involves utilizing digital technologies to reshape an innovative business model for dealing with the new business environment and outperforming their competitors [35]. Digital transformation refers to firms’ transformation process breaking business traditions and seeking innovation and changes based on exploiting digital technologies, including big data, cloud computing, and social platform [36]. For example, past studies have found that digital transformation encourages firms to integrate digital technologies with firms’ operations, in which customers also participate in innovation activities via digital technologies [37]. A social platform named Mi Global Home was built by Xiaomi Technology, which not only promotes communication between users, but also creates an opportunity to understand users’ innovative demands and real feedback for Xiaomi Technology [36].

However, in this context, we focus on the function of digital transformation in coping with the business environment changes caused by the COVID-19 pandemic. In this global crisis, the introduction of digital transformation to overall business activities is more profound than in most of the past [38], because digital transformation plays a crucial role in responding to the COVID-19 pandemic [39]. It helps firms adapt to the changing environment, save costs and own flexibility [40]. In other words, it eliminates the block of space and time, which allows firms to exploit external and internal resources to operate business activities [41]. For instance, digital transformation can improve promotional activities with a falling total expense [21]; digital transformation enables employees to work from home to keep physical a distance when the area where the office is located is temporarily blocked [42]; Microsoft designed a cloud-based platform, known as Azure, that enable healthcare providers to acquire related medical resources quickly [39]; education sector also offers online courses for avoiding potential infection risk among students [43]. Thus, we hypothesize:

**Hypothesis 2.** *Digital transformation is positively related to firms’ performance during the COVID-19 pandemic*.

Competitive pressure has long been seen as a driver for the usage of digital technology, as firms are forced to adapt innovations in order to maintain or seek competitive advantage [19]. Competitive pressure is defined as peer pressure on adopting new technologies [31,32]. Many previous studies have confirmed the positive relationship between competitive pressure and digital transformation, like cloud computing [25], Internet-based selling, service, procurement and coordination [19]. However, it should be mentioned that a study about using Customer Relationship Management (CRM) found that firms’ competitive pressure has a significant and negative effect during the CRM adoption stage [7]. Thus, there is still a slight debate between competitive pressure and firms’ digital transformation. According to the TOE framework, environmental contexts were reported as significant drivers of adopting information systems [7]. A study from the Portuguese hospitality industry found that 92% of 51 hotel managers agreed that the pandemic promoted their digital transformation [43]. The outbreak of COVID-19, coming with pandemic prevention policies, created an uncertain environment, decreasing business opportunities and intensifying competition among firms. In order to gain more competitive advantages in the competition, firms introduce digital transformation. So, we propose the following hypothesis:

**Hypothesis 3.** *Competitive pressure is positively related to digital transformation during the COVID-19 pandemic*.

Digital transformation is a support system to help firms respond positively to increasing competitive pressure and gain more revenue [44]. With the growth of competitive pressure in an uncertain environment, firms attempt to exploit digital transformation to lower operational expenses and raise outreach of their products and services [45]. Furthermore, digital transformation enables firms to energetically use existing resources in the pandemic, such as working remotely and in cloud meetings. These firms who adopt digital transformation can outperform their competitors [19], and survive in the uncertain environment of the COVID-19 pandemic. Thus, we present the following hypothesis:

**Hypothesis 4.** *Digital transformation will mediate the relationship between competitive pressure and firms’ performance during the COVID-19 pandemic*.

### 2.3 Dynamic capabilities theory

The dynamic capabilities theory was developed in strategic management as it offered theoretical direction into firms’ distinct capabilities in a shifting environment [46]. Dynamic capabilities correspond to the comprehensive capabilities to establish, integrate and reconfigure external and internal resources for dealing with a rapidly changing environment [47]. Firms’ dynamic capabilities are considered strategic operation, which enables firms to adapt to the new environment as the opportunity, public crisis or new demand arises [20]. On the other hand, the emerging complex environment represents a platform for firms to show the full potential of their dynamic capabilities [17]. Prior studies displayed the application of dynamic capabilities theory in exploring firms’ strategic responses under unstable business environments. For example, data collected from 274 international firms confirmed that information technology-enabled dynamic capabilities facilitated firms’ competitive performance in uncertain environments [48]; evidence from 339 Chinese firms found that digital transformation helped firms reconstruct internal and external resources under the perspective of dynamic capacity for enhancing organizational resilience [49]; research based on 1162 firms in Nigeria revealed that mobile apps usage enabled firms to maximize opportunity via dynamic capabilities in an innovative environment [46].

In the background of the COVID-19 pandemic, dynamic capabilities are viewed from three dimensions: absorptive, adaptive, and innovative capability [16]. Although it is expected that digital transformation can improve firms’ plights in a public crisis [46], firms with dynamic capabilities are more likely to identify and capture the advantages of digital transformation and apply it in a new environment. Firms with more vital dynamic capabilities can absorb new technology and skills, adapt to uncertain environments and create new opportunities, gaining an impetus for achieving better performance in the COVID-19 pandemic [17].

### 2.4 The moderating effect of absorptive capability

The absorptive capability is an ability that helps an organization acquire and assimilate intangible resources, including knowledge, technology and expertise, then transform and exploit them to generate unique knowledge, technology and expertise [20]. Past scholars have reported that these firms with access to the same quantity of external knowledge might progress at different levels, because their abilities to identify and transform knowledge are different [50]. As a result, the quantity and amount of external knowledge are unevenly distributed in the whole industry, which means that absorptive capability can be viewed as an organization’s competitive advantage [51]. In this context, digital transformation is considered a firm’s dynamic resource from the perspective of dynamic capabilities theory, being an available policy for reconfiguring firms’ resources [49]. And, according to dynamic capabilities theory, the absorptive ability to integrate external and internal resources is a powerful tool for firms to cope with an uncertain environment [52]. Thus, in this context of the COVID-19 pandemic, the value of digital transformation and its effect on firms’ performance depends on their owned characteristics including absorptive capability [53], because the new knowledge and technology acquired from internal (e.g., within the firm) and external (e.g., market) environment might contain context-dependent features, particular factors or specific prerequisites [54]. In other words, digital transformation might improve firms’ performance when firms possess the proper capabilities to absorb and use the new knowledge and technology. For example, firms with a higher degree of absorptive capability can build an effective learning process and a more diverse knowledge base, increasing the potentiality that firms value and positively use new knowledge and technology [55]. Furthermore, the moderating effect of absorptive capability has been confirmed in the field of innovative activities [53], science-to-industry technology transfer projects [56], new product introduction [13] and international venturing [57]. Therefore, the following hypothesis is proposed:

**Hypothesis 5.** *Absorptive capability moderates the relationship between digital transformation and firm performance during the COVID-19 pandemic*, *such that the relationship will be stronger at a higher level of absorptive capability than at a lower of absorptive capability*.

### 2.5 The moderating effect of innovative capability

The innovative capability is defined as an ability to produce, accept and execute fresh ideas and new solutions for responding to risks in the market [58,59]. These innovative ideas and solutions are related to new process design, new service or product development and renovation, which focuses on dealing with the competition in a dynamic and uncertain environment [60]. Innovation is considered a source where an organization develops a competitive advantage, which has been widely explored in large firms across developed countries since 1980 [59]. And, fewer studies paid attention to these firms from emerging economies [61]. Innovative capability development is an investment in firms’ future performance, supplementing firms’ crisis readiness [62]. According to dynamic capabilities theory, both innovative and absorptive capabilities are significant in grasping competitive advantages for firms [63]. Innovative capability can be viewed as complementary to absorptive capability, providing firms with better usage of the knowledge and technologies acquired by absorption [64]. Thus, it is valuable to examine the effect of innovative capability in Chinese firms from the service industry during the COVID-19 pandemic.

In this context, innovative capability has the potential to help firms to respond rapidly and efficiently to uncertainty in the market [65], especially in this global pandemic. For example, firms with better innovation maintain stronger stability in a changing environment, and they can reliably deliver new quality products and services to the market faster, more frequently, and lower cost than their competitors [66]. Innovation allows firms to improve as the changes in their environment [67]. In other words, innovation is a solution to adapt to the uncertainty of the pandemic for firms, as digital transformation works. Firms with higher innovative capability can better grasp new opportunities and apply new knowledge and technology, which will augment firms’ performance in the COVID-19 pandemic. Based on these arguments, the hypothesis is proposed:

**Hypothesis 6.** *Innovative capability moderates the relationship between digital transformation and firm performance during the COVID-19 pandemic*, *such that the relationship will be stronger at a higher level of innovative capability than at a lower of innovative capability*.

### 2.6 The moderating effect of adaptive capability

The adaptive capability refers to an ability to reconfigure resources and coordinate processes for new product and service development [68]. A firm’s adaptive capability involves a dynamic interaction of market, technology and business process, enabling firms to achieve competitive advantages under a turbulent environment [69]. On the other hand, adaptive capability displays the efficacy of problem-solving and the speed of customer response [15]. As dynamic capabilities theory mentioned, dynamic capabilities will bring changes in firms’ resources and capabilities to create sustainable competitive advantage [46]. This capability allows firms to become more flexible in developing beneficial transformations in order to make great progress in a dynamic environment. For example, employees from these firms with adaptive capability can learn various skills and deal with challenges with greater discretion [70]. In this context, adaptive capability is beneficial to firms’ digital transformation. It is an uncertain and highly competitive business environment during the COVID-19 pandemic, which means most employees are unwilling to participate in high-risk activities such as digital transformation in this period [71]. But, a high level of adaptive capability reflects this situation that all employees related to digital transformation can load the required information, knowledge and technology in order to minimize risk in an uncertain environment [14]. Therefore, adaptive capability facilitates the successful conversion of digital transformation into improved firm performance. So, we hypothesize:

**Hypothesis 7.** *Adaptive capability moderates the relationship between digital transformation and firm performance during the COVID-19 pandemic*, *such that the relationship will be stronger at a higher level of adaptive capability than at a lower of adaptive capability*.

**Fig 1:**
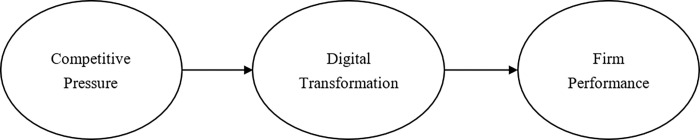
The research model in Study 1.

**Fig 2:**
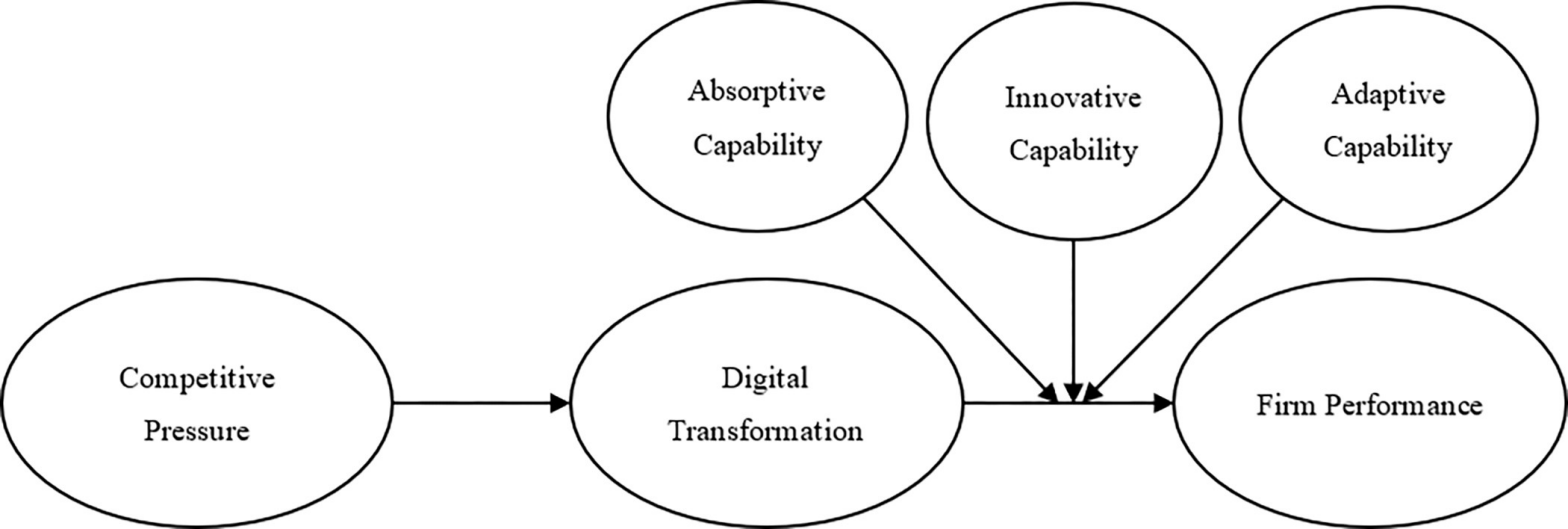
The research model in Study 2.

Based on the TOE framework, we proposed the research model in Study 1 (see Fig 1) in order to explore the effectiveness of digital transformation in Chinese SMEs from the service industry as a strategic response to the COVID-19 pandemic. Then, combined with the TOE framework and dynamic capabilities theory, we proposed the research model in Study 2 (see Fig 2) in order to examine the roles of absorptive capability, innovative capability and adaptive capability in the process of digital transformation, as the strategic role of digital transformation in large Chinese firms from the service industry in the COVID-19 pandemic.

## 3. Methodology

This study investigated the mediating effect of digital transformation as a strategic response to the COVID-19 pandemic. To expand the generalizability and justification of research results, we sought to execute two empirical analyses based on two data sets collected from small- and medium-sized enterprises (SMEs) and large enterprises, respectively. Due to the defect of accurate and reliable accounting in Chinese SMEs [72], Study 1 on SMEs relied on senior managers’ or owners’ subjective evaluations and perceptions of performance instead of the objective indicators to measure firms’ characteristics. On the other hand, we lacked the resources to access enough senior managers or owners from large firms and distribute questionnaires, leading to exploiting data from listed firms’ databases, the China Stock Market and Accounting Research (CSMAR), to measure constructs in this research model. Thus, as suggested by Tang & Yang [73] and Raithel & Hock [74], we used the data collected from the survey in Study 1, while we exploited the data adopted from CSMAR in Study 2. Even though variables were measured by two data sets, corresponding measurements have received theoretical support from previous studies and ensured the justification of methodology [7,20,21,75–77].

Regarding the ethics issue in Study 1, every respondent was briefed on the purpose of the research and informed that all data would be strictly confidential and used for academic purposes only. The relevant information mentioned above was stated at the beginning of the questionnaire to ensure that respondents know their rights and agree with this survey. Thus, this survey obtained written consent from all participants. Finally, this project that collected the data from questionnaires was approved by the Institutional Review Board of the Macau University of Science and Technology. An Ethic Issue Form offered by Macau University of Science and Technology was signed and submitted to promise this article’s authenticity and compliance with academic ethics.

### 3.1 Samples and measures in Study 1

Study 1 explored the effectiveness of digital transformation strategy among Chinese SMEs from the service sector during the COVID-19 pandemic. The data in Study 1 was collected based on a cross-sectional survey executed in China. The construct of competitive pressure was measured by items from Cruz-Jesus et al., Thong, and Zhu et al. (2006) [7,19,30]. The variable of digital transformation was measured by questions from Cruz-Jesus et al., Thong, and Zhu et al. [21,36,49]. The construct of firm performance was developed by Singh et al. and Wang et al. [21,78]. These studies explored the influence of competitive pressure on the adoption of information systems and the effect of information systems on organizational outcomes, which shared the same research background as our study. In summary, the scale used in this research was developed, examined, accepted, and supported by past studies. All items in this survey were initially shown in English. Through the back-translation approach, they were translated into Chinese to minimize translation bias [79]. A pilot test including 10 questionnaires (Chinese edition) was executed among 5 owners of SMEs, 3 professional researchers from the field of organizational behavior and 2 bilingual doctoral students majoring in English and Mandarin, which suggested all Chinese items are clear and understandable in the Chinese context. In April 2022, the survey was distributed to owners or senior managers from SMEs (with less than 500 employees) via social media, including WeChat and E-mail [17,80]. The participants were Chinese students majoring in Executive Master of Business Administration (EMBA), Master of Business Administration (MBA) and Executive Education (EE).

544 valid responses were collected from 1000 SMEs, accounting for 54.4% of the samples. In order to explore the effect of digital transformation in the COVID-19 pandemic, 544 usable samples are qualified as they started their businesses before the pandemic. Estimating the minimum sample size is one of the most significant problems in PLS-SEM (Partial Least Square-Structural Equation Modelling). The ten times rule for calculating the minimum sample size for PLS-SEM has been widely adopted in past studies, but some experts argued that the ten times rule produced inaccurate estimates [81]. Thus, an inverse square root method was recommended to estimate the minimum sample size in PLS-SEM [82]. As suggested by Kock and Hadaya’s [81] inverse square root method, the minimum sample size in this study is 56, which is significantly lower than the 544 samples in Study 1. Thus, 544 is a valid sample size for Study 1. As displayed in Table 2, 21.0% of respondents are SME owners, and the rest are senior managers of SMEs. Both of them were able to judge their SMEs’ status, including perceived competitive pressure, digital transformation and performance [53].

**Table 2:** Respondent profiles.

Measure	Items	Frequency	Percent (%)
Respondent’ level	Owner Senior manager	114 430	21.0 79.0
Firm age	3–4 years 5–6 years 7–8 years 9–10 years Over 11 years	147 200 129 12 56	27.0 36.8 23.7 2.2 10.3
Number of employees	1–50 51–250 251–300 301–500	46 208 189 101	8.5 38.2 34.7 18.6
Annual turnover	Less than 1 million RMB 1–5 million RMB 5–10 million RMB 10–30 million RMB 30–50 million RMB 50–120 million RMB 120–300 million RMB	136 115 98 68 48 49 30	25.0 21.1 18.0 12.5 8.8 9.0 5.5
Location	Eastern China Northern China Northeastern China Central China Southern China Northwestern China Southwestern China	110 103 103 111 41 43 33	20.2 18.9 18.9 20.4 7.5 7.9 6.1
Service industry	Catering Tourism Hospitality Education and training Retail trade Advertising and communication media Consulting and insurance Leasing and business services Culture, sports, and entertainment Health and social work Information technology	40 42 52 38 58 49 41 66 52 44 62	7.4 7.7 9.6 7.0 10.7 9.0 7.5 12.1 9.6 8.1 11.4

All variables were measured via multiple indicators in a self-reported questionnaire with seven-point Likert scales, ranging from 1 (strongly disagree) to 7 (strongly agree). Table 3 shows that all items in the questionnaire were adapted from prior studies. With the assistance of the statistical package SmartPLS, the PLS-SEM was used to assess and evaluate the research model via the PLS algorithm and bootstrapping method for accurate causal-predictive analysis in Study 1 [83,84].

**Table 3:** Measurement, indicator loading, reliability and convergent validity.

Construct	Item	Loading	*t*-value	CR	AVE
Competitive pressure [7,19,30]	After the Covid-19 pandemic outbreak, your firm is under pressure from competitors to adopt digital transformation. After the Covid-19 pandemic outbreak, some of your competitors have already started digital transformation. After the Covid-19 pandemic outbreak, percentage of competitors in your industry that have conducted digital transformation is high. After the Covid-19 pandemic outbreak, percentage of competitors in your industry that have conducted Internet-based selling or servicing is high. After the Covid-19 pandemic outbreak, the level of rivalry among businesses in the same industry is fierce.	0.784 0.813 0.796 0.753 0.751	49.173 52.841 45.654 37.841 39.211	0.886	0.608
Digital transformation [21,36,49]	After the Covid-19 pandemic outbreak, many new business processes built on technologies such as big data, cloud, mobile and social media platform. After the Covid-19 pandemic outbreak, the digital technologies such as social media, big data, cloud and mobile technologies are integrated to drive change. After the Covid-19 pandemic outbreak, the business operations are shifting toward making use of digital technologies such as big data, cloud, mobile and social media platform. After the Covid-19 pandemic outbreak, your firm develops digital products and services. After the Covid-19 pandemic outbreak, supporting communication of commercial and business information through digital technologies (such as big data, cloud computing, mobile and social platforms).	0.764 0.736 0.706 0.757 0.742	36.801 29.317 34.774 35.287 33.000	0.859	0.550
Firm performance [21,78]	After the Covid-19 pandemic outbreak, the growth is more as compared to competitors. After the Covid-19 pandemic outbreak, customer satisfaction of your company is better than that of key competitors. After the Covid-19 pandemic outbreak, quality development of your company is better than that of key competitors. After the Covid-19 pandemic outbreak, responsiveness of your company is better than that of key competitors.	0.819 0.800 0.843 0.738	56.017 49.031 73.074 39.623	0.877	0.642

**Notes:** Loading: Indicator Loading, CR: Composite Reliability, AVE: Average Variance Extracted.

Regarding the issue of common method bias (CMB), according to the direction of Podsakoff et al. [85], this research adopted some approaches to decrease and test CMB. First, the survey data is strictly confidential, ensuring participants respond honestly. Second, we used the full collinearity assessment method [86,87]. When all constructs were regressed on a random dependent variable, all variance inflation factors of constructs were lower than the threshold of 3.3, supporting the data can be considered free of common method bias. Third, we exploited the correlation matrix procedure to examine common method bias [88]. According to the correlation matrix procedure, the values of correlations between variables were 0.782 to 0.832, which were lesser than the threshold of 0.9. Hence, common method bias was not a problem in this study.

### 3.2 Data analysis and results in Study 1

As shown in Table 3, this study evaluated indicator reliability, composite reliability, convergent validity and discriminant validity in constructs. First, to examine the indicate reliability, all indicators’ outer loading values were calculated and were higher than the threshold number of 0.7 with significance [89]. Then, all constructs’ composite reliability (CR) were greater than 0.7, assuming decent degrees of reliability and internal consistency [90]. Besides, all constructs’ average variance extracted (AVE) values were above 0.5, which also demonstrated convergent validity, as it can be deduced that the latent variable explains over 50% variance of related indicators [90]. Fourth, all loadings of indicators were greater than all related cross-loading in Table 4, supporting good levels of discriminant validity between constructs [91].

**Table 4:** Loadings and cross-loadings.

	Competitive pressure (CP)	Digital transformation (DT)	Firm performance (FP)
CP1	**0.784**	0.631	0.664
CP2	**0.813**	0.670	0.656
CP3	**0.796**	0.723	0.647
CP4	**0.753**	0.600	0.574
CP5	**0.751**	0.613	0.636
DT1	0.639	**0.764**	0.587
DT2	0.625	**0.736**	0.582
DT3	0.597	**0.706**	0.583
DT4	0.621	**0.757**	0.579
DT5	0.600	**0.742**	0.567
FP1	0.655	0.636	**0.819**
FP2	0.662	0.658	**0.800**
FP3	0.712	0.672	**0.843**
FP4	0.577	0.527	**0.738**

This study further executed the PLSpredict procedure to evaluate the out-of-sample predictive relevance [92]. As displayed in Table 5, all endogenous indicator values of Q2predict were greater than 0, implying the superiority of research model is over than a naïve prediction and owning predictive relevance [93]. Furthermore, most of values of the root mean squared error (RMSE) in the PLS model were less than those in the liner regression model (LM). And, the PLS model produced lower prediction errors, because three of four Q2predict indicators from the PLS model were over those from the LM [93]. Thus, it can be concluded that this research model in Study 1 is a satisfactory model, as the PLS-based prediction showed a more accurate out-of-sample predictive power.

**Table 5:** PLSpredict assessment PLS vs. LM.

	PLS		LM		(PLS-LM)	
	RMSE	Q^2^predict	RMSE	Q^2^predict	RMSE	Q^2^predict
Firm performance 1	1.166	0.424	1.173	0.418	-0.007	0.006
Firm performance 2	1.117	0.435	1.123	0.428	-0.006	0.007
Firm performance 3	1.241	0.501	1.249	0.493	-0.008	0.008
Firm performance 4	1.521	0.327	1.517	0.331	0.004	-0.004

**Notes:** RMSE: Root mean squared error; MAE: Mean absolute error; PLS: Partial least squares path model; LM: Linear regression model; Number of folds = 10 subgroups; Number of repetitions = 10.

This study executed some assessment procedures to evaluate the outcomes of structural model including multicollinearity between constructs and relevance of the path coefficients. First, the variance inflation factors (VIF) were estimated to exam multicollinearity between constructs. All latent variables’ VIF values were lower than 5, which indicated there was no concerns of multicollinearity among constructs ([89]. Moving forward, we examined path coefficients and t-statistics levels under the bootstrapping method with 5000 samplings. We ran a test of control variables including industry (β = -0.007; bootstrap confidence interval (-0.051, 0.038)), number of employees (β = 0.014; bootstrap confidence interval (-0.032, 0.059)), location (β = -0.009; bootstrap confidence interval (-0.059, 0.040)), firm age (β = -0.015; bootstrap confidence interval (-0.061, 0.027)), and annual turnover (β = 0.007; bootstrap confidence interval (-0.038, 0.053)), which reported control variables have no significant effects [7,17,20].

**Fig 3:**
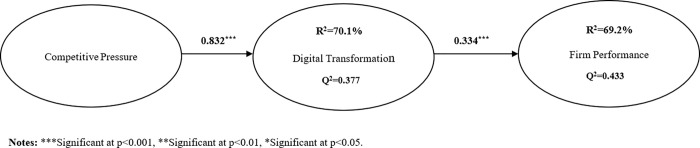
Structural model results in Study 1. Notes: ***Significant at p<0.001, **Significant at p<0.01, *Significant at p<0.05.

From Fig 3, it can be viewed that the research model explained 70.1% of the variation in digital transformation, as 69.2% of the variation in firm performance. It also can be seen that their blindfolding-based Q2 metric values were greater than 0, supporting a decent predictive relevance [91]. As shown in Table 6, it demonstrated that competitive pressure positively, directly and significantly influenced digital transformation (ß = 0.832) and firm performance (ß = 0.536). Therefore, H1 and H3 were supported. And also, it illustrated that digital transformation positively and significantly influenced firm performance (ß = 0.334), thus, H2 was supported.

**Table 6:** The assessment of structural model.

Relationship	Std Beta	Std error	*t-*value	*p-*value	Bootstrap confidence interval		*f* ^2^
Competitive pressure -> Firm performance	0.536	0.041	12.994	0.000	0.453	0.615	0.292
Competitive pressure -> Digital transformation	0.832	0.015	53.925	0.000	0.801	0.860	2.250
Digital transformation -> Firm performance	0.334	0.041	8.112	0.000	0.255	0.416	0.114
Competitive pressure -> Digital transformation -> Firm performance	0.278	0.035	7.895	0.000	0.212	0.349	----

**Notes:** Bootstrapping based on n = 5,000 bootstrap samples; ***Significant at p<0.001, **Significant at p<0.01, *Significant at p<0.05 (two-tailed test).

Then, a mediating effect test was executed (see Table 7). Followed a formal mediation analysis [93,94], the indirect effect between competitive pressure and firm performance was tested and was significant. The second step was that the direct effect of competitive pressure on digital transformation was estimated and showed significance. Lastly, the direct effect shared the same direction as the indirect effect. Thus, it can be conducted that digital transformation had a partial mediating effect between competitive pressure and firm performance, which meant H4 was supported.

**Table 7:** The mediating effect test.

Total effect			Direct effect			Indirect effects on performance					
	Path	*p* value		Path	*p* value		Point estimate	Confidence interval		Sig.	VAF
CP -> FP	0.814	0.000	CP -> FP	0.536	0.000	CP -> DT -> FP	0.278	0.212	0.349	Yes	34.15%

**Notes:** CP -> FP: Competitive pressure -> Firm performance; CP -> DT -> FP: Competitive pressure -> Digital transformation -> Firm performance; Sig.: Significance; VAF: Variance Accounted For.

### 3.3 Samples and measures in Study 2

Study 1 confirmed the effectiveness of the digital transformation strategy among Chinese SMEs during the COVID-19 pandemic. According to Tang et al. [73], Study 1 was beneficial in building the baseline relationship between competitive pressure and firm performance via digital transformation. Therefore, in Study 2, we confirmed the mediating effect of digital transformation in the relationship between competitive pressure and firm performance. Also, in Study 2, based on the baseline relationship, we explored the moderating effect of dynamic capabilities on the relationship between digital transformation and firm performance to enhance the theoretical contributions of our findings.

Our study 2 panel data sample covered Chinese listed firms on the Shanghai and Shenzhen Stock Exchanges from 2007–2020. In this study, we exploited the data of listed companies from service industry as the data of large companies from service industry (more than 500 employees) collected from the China Stock Market and Accounting Research (CSMAR) database [95]. Data from CSMAR contains comprehensive historical information of listed firms on the Shanghai and Shenzhen Stock Exchanges like financial statements and corporate governance reports, which have been applied widely in studies related to corporate strategy [95,96]. The year of 2007 was considered as the starting point, because Chinese listed firms have been required to adopt new accounting standards since 2006. And, the year of 2020 was viewed as the ending point, because CSMAR database currently discloses data up to 2020.

This Study 2 excluded B-share firms because they only disclosed financial data in their annual reports without relative data required by Study 2. Besides, it excluded firms that have been listed for one year or less in order to show the influence of the COVID-19 pandemic and reduce the uncertainty from newly listed firms. Moving forward, it dropped firm-year observations with missing values and obtained a final sample of 31859 firm-year observations from 4341 listed companies.

### 3.4 Dependent variable in Study 2

Tobin’s Q served as the dependent variable in this study. Tobin’s Q as a metric has won widespread acceptance because it was claimed to be forward-looking and comparable across diverse industries. Tobin’s Q has been widely used to measure firms’ financial performance. It is defined the Q ratio as a firm’s market value relative to its assets’ replacement cost [97]. Researchers have exploited the Q ratio to study the effects of market power on performance [75,76]. In this study, we used Chung and Pruitt’s method to calculate Q ratio [97]. The advantage of this approach is that the financial and accounting information required by this formula is available in the CSMAR database.

### 3.5 Explanatory variable in Study 2

The Herfindahl-Hirschman Index (HHI) is a measure of the industry’s competitiveness in a given year. Drawing on the measures of Haveman et al. and Jia et al., the HHI among service industry segment for the year was used to measure the degree of competitiveness [77,98]. The HHI is calculated by classifying each service industry segment based on the industry code (two digits) of the Securities and Futures Commission, then calculating the market share of each company according to the business revenue of all companies in each segment, and finally calculating the squared sum of the market shares of all companies in the segment. Whit less the HHI, relative service industry presents a stronger competitive pressure. For a more intuitive interpretation of the subsequent empirical results, the index is processed (1-HHI), to obtain the industry competitiveness index (IH) for the model test in this paper.

### 3.6 Mediating variable in Study 2

Prior studies have established various measures on firms’ digital transformation (DT), which represented the degree of firms’ digital transformation [75]. With the rapid development of machine learning and text mining technology, Study 2 attempted to construct the DT index of listed firms by text mining. During the process of collecting data about the DT index, this study firstly retrieved and collated the annual reports of sample firms via the Python crawler function and secondly extracted all textual contexts via Python program to establish a data pool for subsequent feature word screening [75]. After that, this study identified the feature words of digital transformation from the academic and industrial perspective. On the one hand, the feature words from the academic perspective were absorbed from influential literature on the field of digital transformation [99,100]. On the other hand, the feature words were grasped from firms’ website. Then the Study 2 created a pool of feature words including digital transformation, big data, cloud computing, blockchain, artificial intelligence and etc. Lastly, based on the pool of feature words of digital transformation, the Study 2 searched, matched and counted the frequency of words after extracting text content from sample firms’ annual reports under the assistant of these tools including Jieba package and the word list of deactivations. The ultimate characteristics of feature words were summarized after all steps above. As the typical characteristic of right deviation owned by the set of data, the Study 2 executed a logarithmic process using In (‘the total frequency’ +1) to measure the digital transformation of firms.

### 3.7 Moderating variables in Study 2

The Study 2 constructed a panel data structure to measure dynamic capabilities in three dimensions: innovation capability, absorptive capability and adaptive capability [101,102]. The specific indicators are as follows.

Innovation capability (IC) in Study 2 used the two indicators of sample firms’ annual R&D investment intensity and the proportion of technical employees for comprehensive evaluation. The values of two indicators were standardized separately and summed to the combined value of innovation capability:

$$\mathrm{IC}=\frac{x_{\mathrm{RD}}-\min_{\mathrm{RD}}}{\max_{\mathrm{RD}}-\min_{\mathrm{RD}}}+\frac{x_{\mathrm{IT}}-\min_{\mathrm{IT}}}{\max_{\mathrm{IT}}-\min_{\mathrm{IT}}}$$

Absorptive capacity (Absor) was measured using R&D expenditure intensity, i.e., the ratio of annual R&D expenditure to operating revenue of sample firms.

Adaptive capability (Adapt) was measured by the coefficient of variation of three types of expenditure on R&D, capital and advertising reflecting the flexibility of resource allocation and showing firms’ adaptive capabilities.

### 3.8 Control variables in Study 2

We controlled several factors that might influence firms’ digital transformation including firm size (Size, the natural logarithm of a firm’s total asset), firm age (Age, counting from the start of firm), the number of board directors (Board, the logarithm of the number of board members), the ratio of independent directors in the whole board directors (Inde, the proportion of independent directors), the CEO and the chairman of board are held by the same person (Dualiy, 1 for the CEO and he chairman of board are held by the same person and zero otherwise), the ownership of the largest shareholder (Top1, the share of the largest shareholder), some financial indicators (Lev, asset liability ratio; Cash, cash flow ratio; Tangiblity, fixed assets ratio), firms’ dummy and years’ dummy.

It can be viewed the measurement scales in Table 8 and reported the means and standard deviations of the variables. In the regression models, this Study 2 computed variance inflation factors (VIFs), which ranged from 1.05 to 1.74 and were far below the cutoff of 10. Thus, multicollinearity was not a major concern.

**Table 8:** Descriptive statistics.

Variables	Obs	Mean	SD	Min	Med	Max
TobinQ	31859	2.2468	1.4542	0.9189	1.7756	9.5098
DT	31859	1.1222	1.3389	0.0000	0.6931	4.9053
IH	31859	0.9426	0.0909	0.5343	0.9826	0.9919
Size	31859	22.0112	1.2482	19.6582	21.8339	25.9283
Age	31859	2.7861	0.3672	1.6094	2.8332	3.4657
Board	31859	2.2525	0.1787	1.7918	2.3026	2.7726
Dualiy	31859	0.2722	0.4451	0.0000	0.0000	1.0000
Inde	31859	0.3730	0.0528	0.3077	0.3333	0.5714
Top1	31859	0.3677	0.1497	0.0842	0.3544	0.7572
Lev	31859	0.4176	0.2047	0.0500	0.4108	0.8919
Cash	31859	0.9674	1.6980	0.0205	0.4032	11.2034
Tangiblity	31859	0.2259	0.1625	0.0037	0.1917	0.7146

This Study 2 also tested these hypotheses using panel analysis with standard errors clustered at firm level. It took several initiatives to address potential endogeneity in this analysis. First, the Study 2 lagged dependent variables by one year to minimize the possibility of reverse causality. Second, the Study 2 controlled a set of executive-, board-, and company-level variables that may affect firm performance, simultaneously. Third, the Study 2 included year and industry fixed effect (i.e., 14 year-dummies, 19 industry-dummies, and 4341 firm-dummies) in the regression models to account for within-group variation over time and limit the potential bias caused by omitted variables. This empirical approach allowed the predicted mean of the dependent variable to vary across groups and thus controlled unobserved heterogeneity [103].

To test research hypotheses, we proposed the regression models as follow:

$${\mathrm{Tobinq}}_{i,t+1}=\alpha_{0}+\alpha_{1}{\times \mathrm{IH}}_{\mathrm{i,t}}+\alpha_{k}\sum {\mathrm{Control}}_{\mathrm{i,t}}{+\varepsilon }_{\mathrm{i,t}}$$ (1)

$${\mathrm{DT}}_{i,t+1}=\beta_{0}+\beta_{1}{\times \mathrm{IH}}_{\mathrm{i,t}}+\beta_{k}\sum {\mathrm{Control}}_{\mathrm{i,t}}{+\varepsilon }_{\mathrm{i,t}}$$ (2)

$${\mathrm{Tobinq}}_{i,t+1}=\gamma_{0}+\gamma_{1}{\times \mathrm{IH}}_{\mathrm{i,t}}+\gamma_{1}{\times \mathrm{DT}}_{\mathrm{i,t}}+\alpha_{k}\sum {\mathrm{Control}}_{\mathrm{i,t}}{+\varepsilon }_{\mathrm{i,t}}$$ (3)

In addition, the Study 2 tested these hypotheses with fixed-effect models estimating the influence of independent variables on firm performance and the integration effect of explanatory variables. To examine the moderating hypotheses, this study executed a main model within moderates and related results were presented and tested via graphs, confidence intervals and robust standard errors.

### 3.9 Data analysis and results in Study 2

Table 9 reported the results of fixed-effect regression and logit regression. Model 1 displayed the main effect of IH on firm performance. The results of Model 2 showed that the estimated coefficient of IH was positive and significant at 5% level, indicating that IH was positively related with firms’ digital transformation. In other words, competitive pressure enhanced the degree of digital transformation among Chinese listed firms. And, the results of Model 3 showed that the IH coefficient was significantly negative, indicating that external industry competitive pressure reduced the level of firm performance, and suggesting that the mediating effect of digital transformation between competitive pressure and firm performance was significant. Thus, H1 was not supported and H2, H3 and H4 were supported.

**Table 9:** The mediating mechanism of relationship between competitive pressure and firm performance.

	Model 1	Model 2	Model 3
	Tobinq	DT	Tobinq
IH	-0.9271***	0.3958**	-0.9659***
	(-3.8618)	(2.1368)	(-4.0170)
DT			0.0489***
			(3.6585)
Size	-0.5165***	0.2083***	-0.5282***
	(-15.3695)	(9.3110)	(-15.6472)
Age	0.7784***	-0.0217	0.7732***
	(5.2059)	(-0.1461)	(5.1874)
Board	-0.1151	0.2298***	-0.1300
	(-0.9857)	(2.7185)	(-1.1133)
Dualiy	-0.0358	0.0164	-0.0358
	(-1.2578)	(0.6739)	(-1.2604)
Inde	0.2879	-0.0258	0.2845
	(0.9769)	(-0.1141)	(0.9655)
Top1	-0.6427***	-0.3247**	-0.6217***
	(-4.0323)	(-2.5431)	(-3.9132)
Lev	0.0722	-0.0872	0.0767
	(0.6420)	(-1.0687)	(0.6830)
Cash	-0.0831***	-0.0292***	-0.0812***
	(-8.5072)	(-4.3319)	(-8.2860)
Tangiblity	-0.1223	-0.4944***	-0.0982
	(-0.8777)	(-5.3183)	(-0.7084)
Constant	11.9605***	-4.8606***	12.2686***
	(12.7308)	(-7.3140)	(13.0785)
Industry	Yes	Yes	Yes
Year	Yes	Yes	Yes
N	26846	26846	26846
Adj.R^2^	0.2667	0.3639	0.2676

**Notes:** ***Significant at p<0.01, **Significant at p<0.05, *Significant at p<0.10, T-value shown in parentheses.

**Fig 4:**
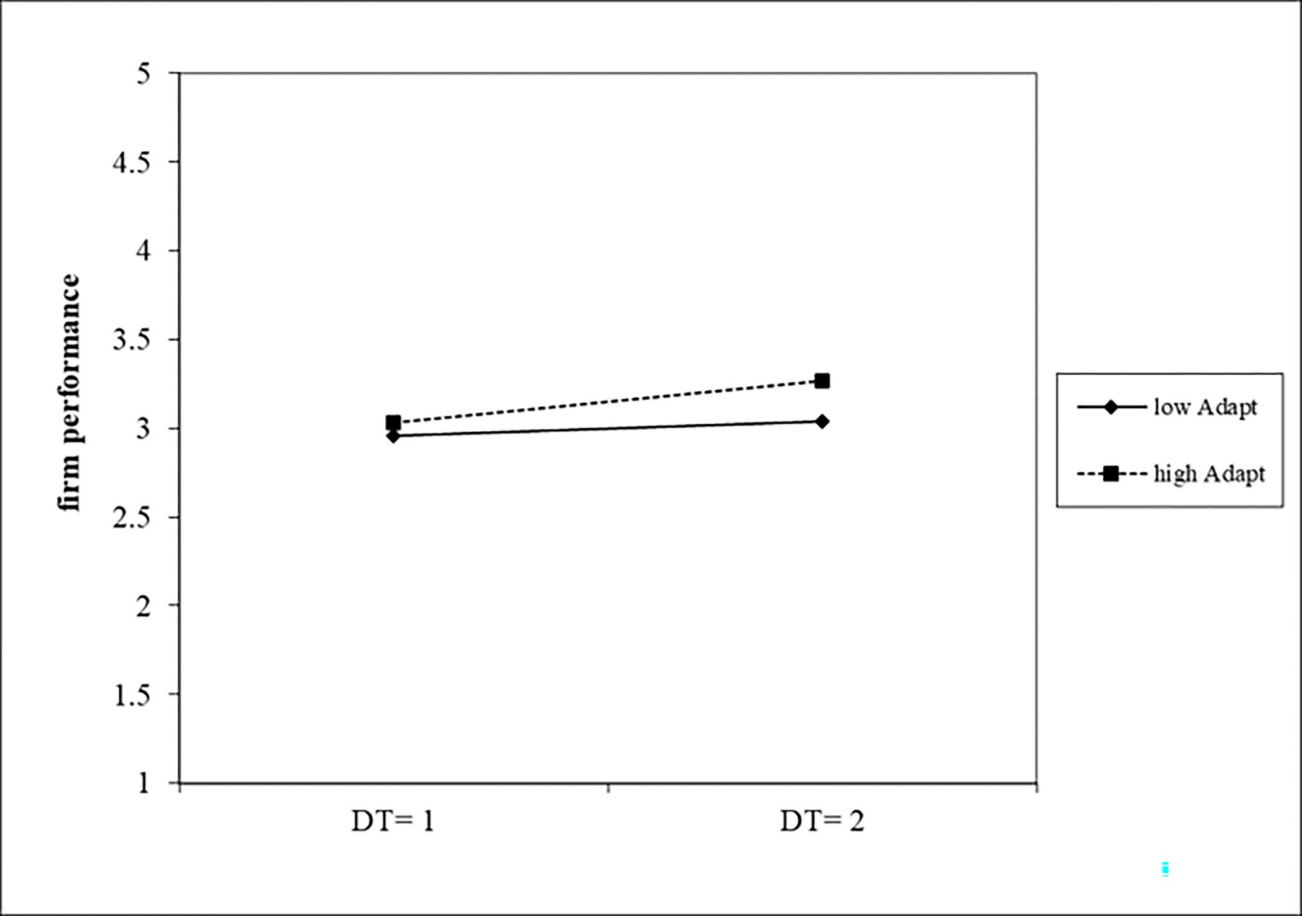
The moderating effect of innovation capability on firm performance.

**Fig 5:**
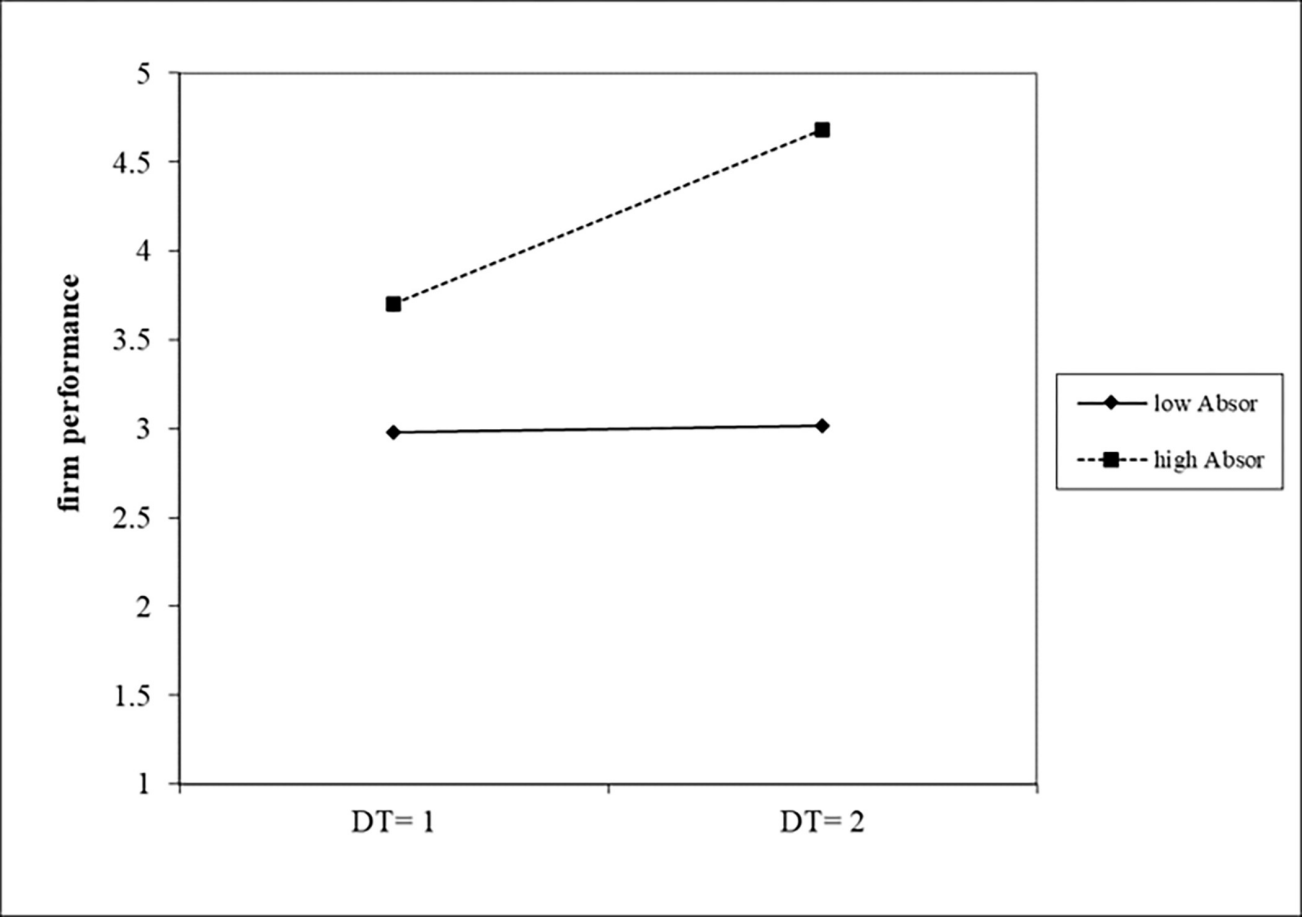
The moderating effect of absorptive capability on firm performance.

**Fig 6:**
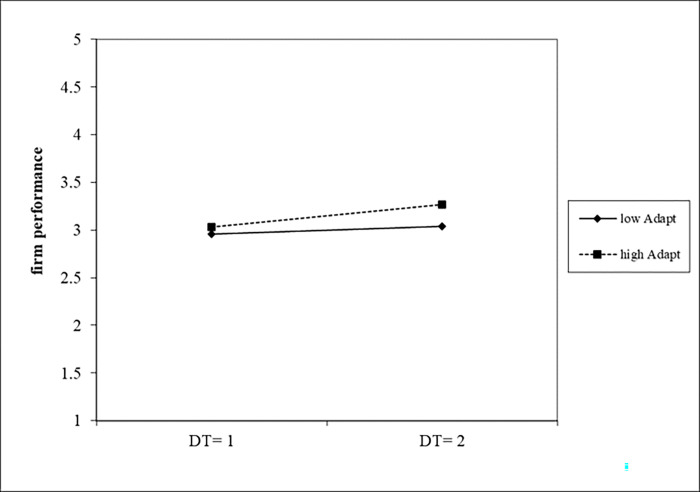
The moderating effect of adaptive capability on firm performance.

Table 10 reported the results of estimating the interaction effects of dynamic capabilities on the relationship between digital transformation and firm performance. In Model 4, we found a positive and significant interaction between digital transformation and innovation capacity (b = 0.0575 p<0.05). We graphed the moderating effect of innovation capacity on the relationship between digital transformation and firm performance in Fig 4. As it shown, high levels of innovation capacity (the dotted line) strengthened the positive relationship between digital transformation and firm performance. Model 5 revealed a positive and significant coefficient estimate for the interaction between digital transformation and absorptive capability (b = 0.4719, p<0.1). We graphed the moderating effect of absorptive capacity on the relationship between digital transformation and firm performance in Fig 5. Similarly, the results of Model 6 showed that the estimated coefficients of digital transformation and adaptive capability were significantly positive (b = 0.0770, p<0.05). We graphed the moderating effect of adaptive capability on the relationship between digital transformation and firm performance in Fig 6. In summary, the relationship between digital transformation and firm performance were positively moderated by absorptive capability, innovative capability and adaptive capability, thus H5, H6 and H7 were supported.

**Table 10:** The moderating effect of dynamic capability.

	Model 4	Model 5	Model 6
	Tobinq	Tobinq	Tobinq
DT	0.0173	0.0183	0.0411***
	(1.2710)	(1.3251)	(3.1403)
IC	0.1010		
	(1.5771)		
DT×IC	**0.0575** **		
	**(2.0593)**		
Absor		1.1913**	
		(1.9868)	
DT×Absor		**0.4719** *	
		**(1.9083)**	
Adapt			0.1466***
			(4.3025)
DT×Adapt			**0.0770** **
			**(1.9933)**
Size	-0.4629***	-0.4596***	-0.6396***
	(-12.6114)	(-12.5364)	(-15.5548)
Age	0.7068***	0.7133***	0.7397***
	(4.5532)	(4.5991)	(5.0373)
Board	-0.0631	-0.0668	-0.1473
	(-0.5170)	(-0.5472)	(-1.2686)
Dualiy	-0.0539*	-0.0534*	-0.0362
	(-1.7703)	(-1.7572)	(-1.2762)
Inde	0.5156	0.5059	0.2405
	(1.6150)	(1.5859)	(0.8289)
Top1	-0.6399***	-0.6455***	-0.6042***
	(-3.5519)	(-3.5799)	(-3.8242)
Lev	0.1658	0.1707	0.0815
	(1.3337)	(1.3691)	(0.7267)
Cash	-0.0766***	-0.0784***	-0.0731***
	(-7.7149)	(-7.9493)	(-7.3627)
Tangiblity	-0.2031	-0.2257	-0.1354
	(-1.3356)	(-1.4764)	(-0.9769)
Constant	9.5935***	9.5397***	14.0899***
	(9.5304)	(9.4717)	(13.2794)
Industry	Yes	Yes	Yes
Year	Yes	Yes	Yes
N	20989	20989	26846
Adj.R^2^	0.2249	0.2251	0.2682

Notes

***Significant at p<0.01

**Significant at p<0.05

*Significant at p<0.10, T-value shown in parentheses.

### 3.10 Further research in Study 2

**Fig 7:**
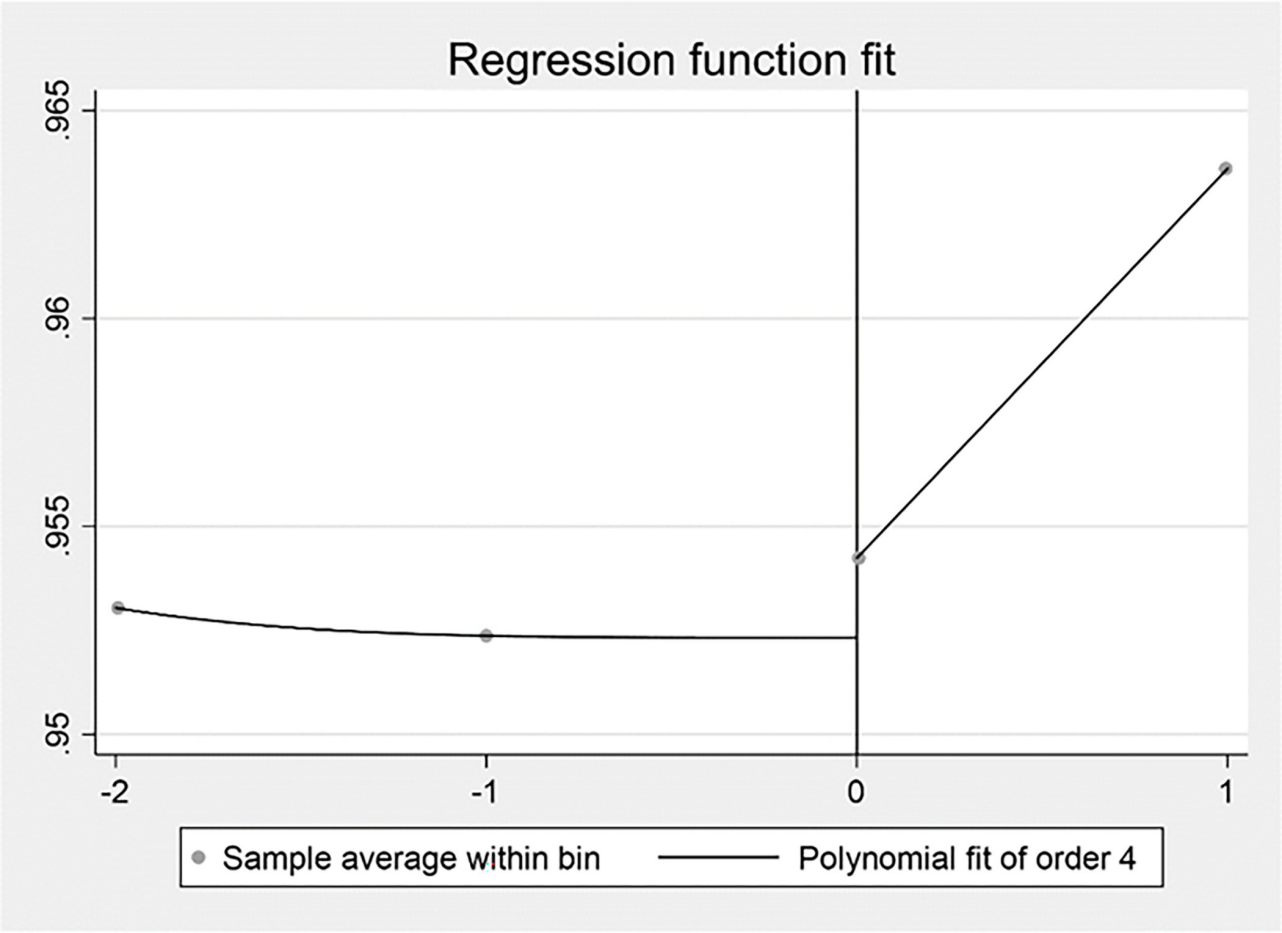
The COVID-19 pandemic and IH.

**Fig 8:**
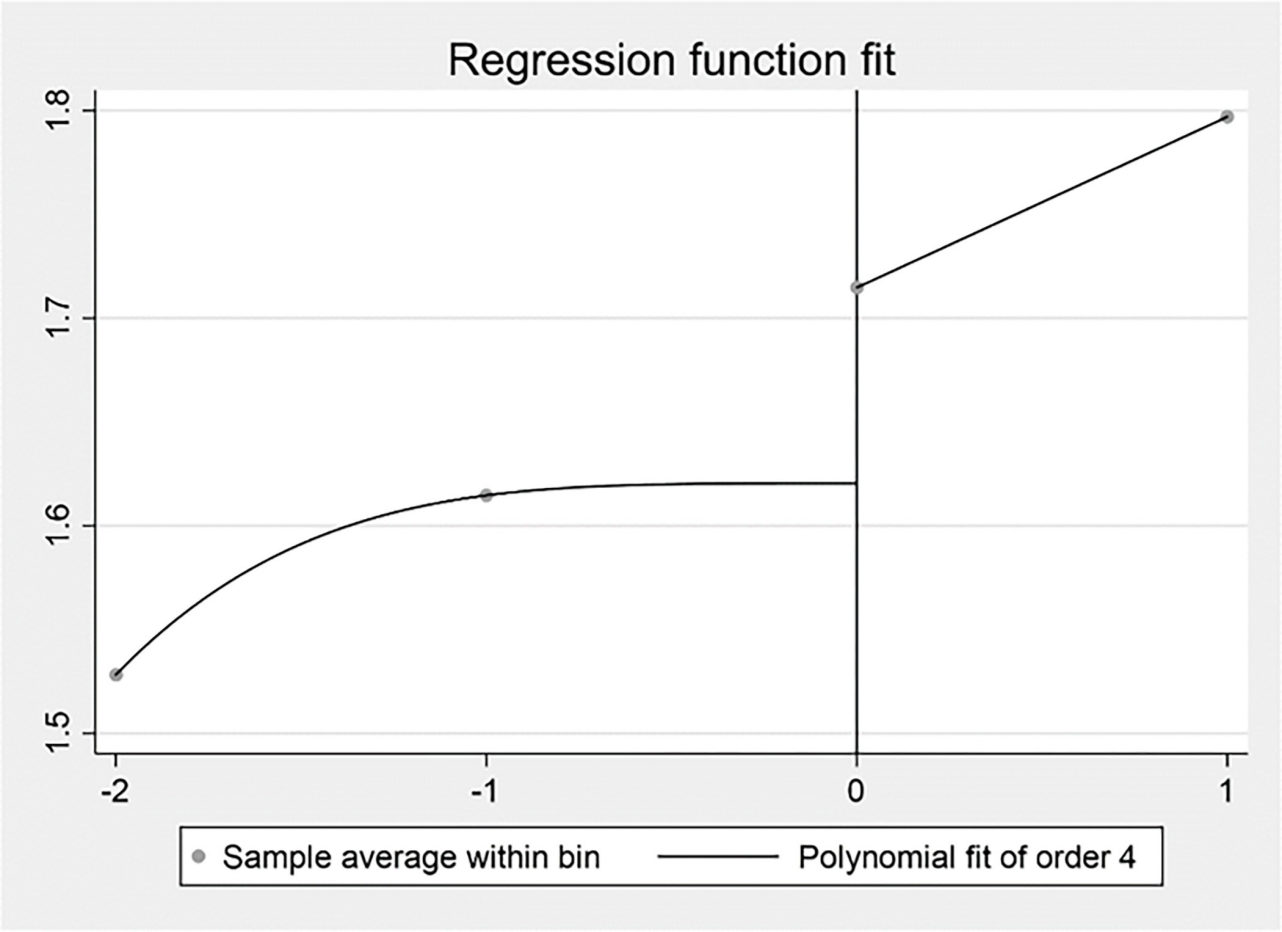
The COVID-19 pandemic and digital transformation.

**Fig 9:**
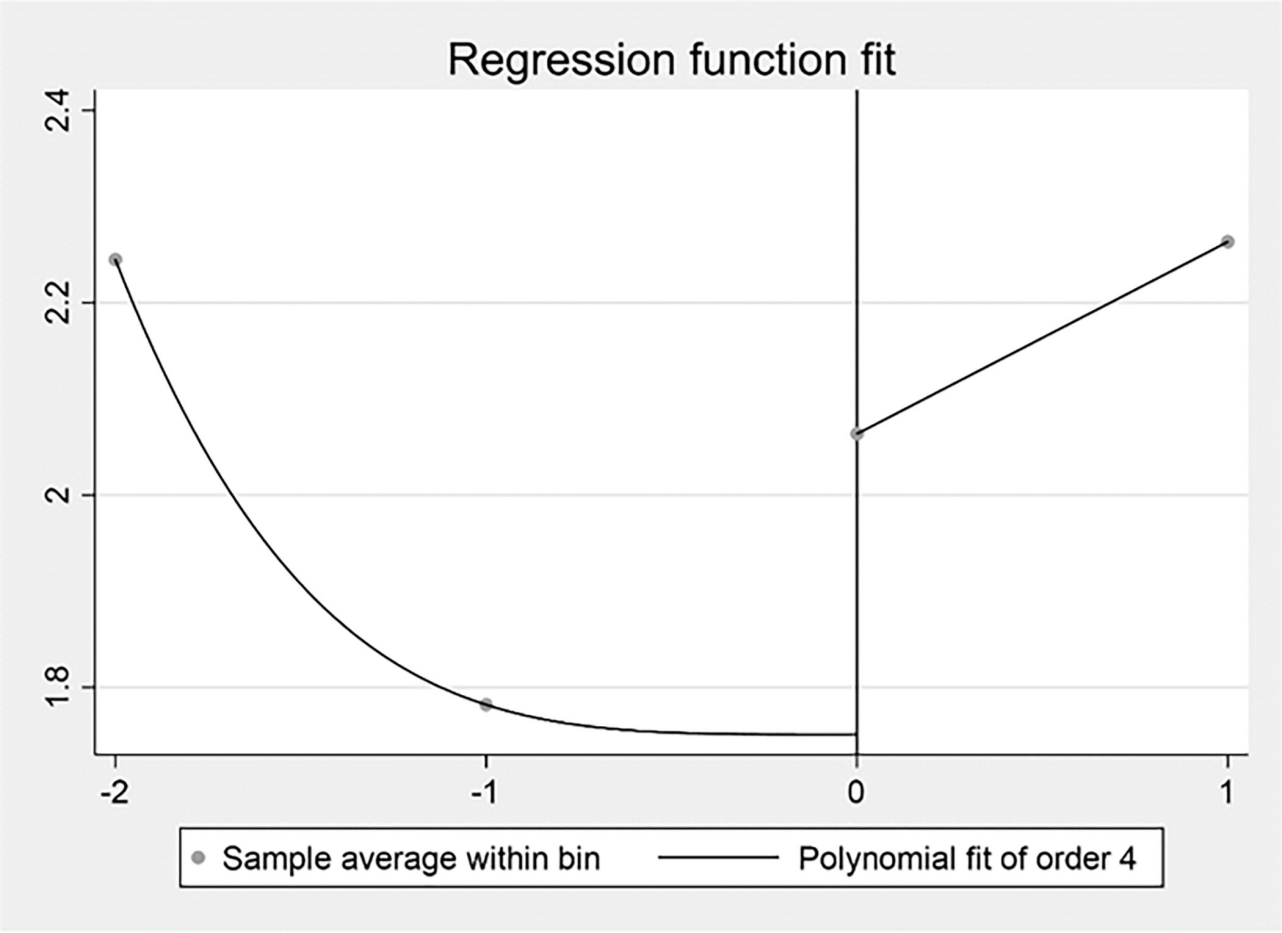
The COVID-19 pandemic and firm performance.

The Study 2 used the Regression Discontinuity Design (RDD) to examine the impact of external competitive pressures on firm performance using the breakpoint time of the outbreak of COVID-19 at the end of 2019. Due to the limitation of data, the Study 2 intercepted data from 2017–2020. The results were displayed by plotting the regression discontinuity. As shown in Figs 7, 8 and 9, there was a significant jump in competitive pressure, digital transformation and firm performance around the year of 2019 under the impact of the COVID-19 pandemic. It confirmed digital transformation improved the negative influence of competitive pressure on firm performance for Chinese listed firms from service industry in the COVID-19 pandemic. Specifically, Fig 7 illustrated that the relationship between the driver variable of the COVID-19 pandemic and IH, which indicated a clear downward jump in the IH ratio to the right of the breakpoint. In Fig 8, it represented the relationship between the COVID-19 pandemic and digital transformation, which showed a clear upward jump in digital transformation near the discontinuity. Fig 9 reported the relationship between the COVID-19 pandemic and Tobin’s Q, and it demonstrated that there was also a clear upward jump in firm performance near the discontinuity. Combined with these figures, the initial indication disclosed the impact caused by the COVID-19 pandemic has an impact on performance by raising competitive pressures and promoting digital transformation.

In Study 2, the optimal bandwidth at minimum mean square error was calculated following the specific method [104] and the regression results for the regression discontinuity were estimated. As shown in Table 11, the optimal bandwidth was 1.1602 years when the dependent variable was IH. The regression discontinuity treatment effect was not significant. When the dependent variable was digital transformation, the optimal bandwidth was 1.8911 years. The regression discontinuity treatment effect was significantly positive at the 1% level. When the dependent variable was Tobin’s Q, the optimal bandwidth was 1.239 years. The regression discontinuity treatment effect was significantly positive at the 1% level. Given the results of the fixed-effect regression and regression discontinuity design, it can be concluded that digital transformation relieved the impact of the COVID-19 pandemic and improved firm performance, which means digital transformation was an effective strategic response to the COVID-19 pandemic for Chinese listed firms from service industry.

**Table 11:** Regression discontinuity results.

	IH	DT	Tobinq
Is the COVID-19 pandemic	0.0019	0.1001***	0.2816***
bandwidth	1.1602	1.8911	1.239
N	13247	13247	13247

Notes

***Significant at p<0.01

**Significant at p<0.05

*Significant at p<0.10.

## 4. Discussion and implications

The COVID-19 pandemic creates huge challenges for most service businesses, as the uncertain social distancing restrictions and cities’ lockdowns decrease people’s consumption demand and consumption opportunities [5]. And, digital transformation has been widely considered a critical path to achieving competitive advantage in response to the public crisis for firms [17]. For example, during the COVID-19 pandemic in China, evidence from catering displayed that the delivery chain, including ordering online and takeaway, avoids physical contact between humans [39]. Evidence from hospitality and tourism exhibited that they mark safe attractions and safe hotels on related mobile applications via an online-offline integration featured function and invest in new high-tech facilities to avoid physical contact between guests and employees [105,106]. Evidence from service employees showed that a virtual work environment built by digital technology guarantees service businesses running when they are advised to adopt safety measures [1]. However, most studies in the field of digital transformation in response to the COVID-19 outbreak have only focused on its outcomes, such as organizational efficiency [107], individual work performance [1] and organizational resilience [49], and ignored the antecedent of digital transformation caused by the COVID-19 outbreak. This quantitative research within two studies comprehensively demonstrates digital transformation’s antecedent and outcome in the service industry.

Based on the TOE framework, the results from Study 1 and Study 2 presented that intense competitive pressure significantly affects firm performance. Interestingly, the directions between competitive pressure and firm performance among SMEs and listed firms differ. In Study 1, the effect size *f* ^2^ value indicated that competitive pressure had a large and positive influence on firm performance among SEMs [89], while in Study 2, the result of fixed-effect regression indicated that competitive pressure had a negative influence on firm performance among listed firms. Both two studies found that digital transformation had a positive influence on firms, including SMEs and listed firms, thus, supporting H2. Similarly, both two studies confirmed that competitive pressure had a positive influence on digital transformation, thus H3 was supported. After testing these direct relationships, Study 1 and Study 2 reported digital transformation mediated the relationship between competitive pressure and firm performance. In other words, competitive pressure positively impacted firm performance via digital transformation for SMEs and listed firms in the COVID-19 pandemic, supporting H4. Furthermore, based on dynamic capabilities theory, Study 2 also deeply explored how digital transformation influence firm performance among listed firms. The results of Study 2 demonstrated that the effect of digital transformation on firm performance was moderated by firms’ absorptive capability, innovative capability and adaptive capability, so H5, H6 and H7 were supported.

### 4.1 Implications for research

This research within two studies is grounded in and contributes to the theoretical perspectives of the TOE framework and the digital transformation literature. It is one of the first empirical studies to understand the antecedent and outcome of digital transformation among Chinese firms from the service industry. First, we identify competitive pressure as a significant antecedent of digital transformation for SMEs and listed firms in the COVID-19 pandemic. Prior studies affiliated with digital transformation during the COVID-19 pandemic have paid considerable attention to the outcomes of firms’ digital transformation [1,17,38,107]. Perceptive as these studies have been, the straightforward question of what factor may promote Chinese service firms’ digital transformation remains understudied in the COVID-19 pandemic. The TOE framework predicted firms’ environmental characteristics are critical determinants of firm behaviors in adopting innovative strategies [22]. Combined with the TOE framework, we supplement the digital transformation research in the COVID-19 pandemic and extend the applied field of the TOE framework in a public crisis by confirming a direct relationship between competitive pressure and digital transformation.

This research also extends our understanding of the relationship between competitive pressure and firm performance. The results from Study 1 and Study 2 reported an opposite and significant relationship between competitive pressure and firm performance. Study 1 presented that competitive pressure positively affects firm performance, which is in line with most past studies [21,108,109]. And, a few studies reported there is no significant relationship between competitive pressure and firm performance [110]. However, Study 2 found that competitive pressure negatively and significantly affected firm performance among Chinese listed firms from the service sector. A study on the adoption behavior of cloud computing in Germany exhibited that compared to SMEs, large firms thought cloud computing was more complex and more challenging to implement [111]. Similarly, a study also found that large firms paid more attention to potential risks than SMEs [112]. Thus, in the COVID-19 pandemic, large firms might more easily perceive stronger competitive pressure than SMEs, harming their performance. Joining the above discussion, this study attempts to answer the debate about the relationship between competitive pressure and firm performance.

Third, our theoretical predictions in Study 1 and Study 2 are confirmed through SEM and the fixed-effect regression. Both two studies demonstrated digital transformation improved firm performance, and there was a positive mediation effect of digital transformation between competitive pressure and firm performance. So, it can be concluded that the digital transformation strategy is a practical strategic response to the COVID-19 pandemic for Chinese SMEs and large firms in the service industry. Two studies with two methods in this research boost our conclusions’ generalizability and guarantee the robustness of our theoretical predictions [73].

Finally, we are grounded in and extend to the theoretical application of the TOE and dynamic capabilities theory, providing evidence of the moderation effects of absorptive capability, innovative capability and adaptive capability in the relationship between digital transformation and firm performance among large Chinese firms from the service industry in Study 2. Most previous studies related to digital transformation have explored the mediation effect of dynamic capabilities [9,17,46]. Insightful as these scholars have been, they failed to test the moderation effects of dynamic capabilities theory on firms’ transformation process. This research further advances the literature by exploring three organizational abilities based on dynamic capabilities theory as three boundary conditions to the effect of digital transformation on organizational outcome. This also distinguishes the present research from studies on dynamic capabilities viewed as the mediating roles or antecedents of organizational behavior [12,48,49]. Thus, as absorptive capability, innovative capability and adaptive capability have been found to influence the relationship between digital transformation and firm performance, Study 2 extends the application of dynamic capabilities theory.

### 4.2 Implications for practice

The results of this research have some practical implications for both SMEs and large firms. First, senior managers and owners in SMEs should have confidence in the storm of the COVID-19 pandemic, and moderate optimism can help them perform better under strong competitive pressure [73]. The results in Study 1 confirmed competitive pressure can promote SMEs to generate better firm performance. Furthermore, according to the results of Study 1 and Study 2, SMEs and large firms should invest more resources in digital transformation, including big data, application usage, cloud technology, etc., to achieve better performance during the COVID-19 pandemic. Third, large firms should effectively develop their dynamic capabilities, enabling them to exploit digital technology better to improve their performance.

### 4.3 Limitations and further research

Certain fields of the outcomes displayed should be interpreted in light of their limitations. First, we collected SMEs’ data using a survey approach and collected large firms’ data using panel data. The operationalization of collecting data from SMEs and large firms should be consistent in future research. Due to the defect of accurate accounting in Chinese SMEs [72], Study 1 on SMEs relied on senior managers’ or owners’ subjective evaluations and perceptions of performance instead of the objective indicators to measure firms’ characteristics. Due to the defect of available resources, Study 2 on large firms adopted data from listed firms’ database CSMAR. Thus, further studies should adopt the same approach. For instance, scholars can ask CEOs or top management from large firms or listed firms directly about their perceived competitive pressure; scholars can find enough SMEs with accurate accounting processes to establish statistical power. Second, although this research referred to the TOE framework to explain the theoretical mechanism among competitive pressure, digital transformation and firm performance, we did not pay more attention to the difference in the relationship between competitive pressure and firm performance in Study 1 and Study 2. Future scholars may consider the potential research gap. Third, we failed to illustrate whether dynamic capabilities have similar functions among SMEs as they work in large firms, which constructs the possibilities of dynamic capabilities in SMEs’ context. Lastly, due to the limitation of the speed of renewing data in CSMAR, Study 2 executed RDD based on the data until 2020, after the outbreak of COVID-19. Future researchers can test the whole model with a richer data set.

## Supporting information

S1 File (PDF)

S2 File (XLSB)
