# Supplementary material for: How firms cope with social crisis: The mediating role of digital transformation as a strategic response to the COVID-19 pandemic
Source: PLoS One. 2023 Apr 4;18(4):e0282854. doi: 10.1371/journal.pone.0282854 (PMC10072485; doi:10.1371/journal.pone.0282854)
Supplement: S1 File — (PDF) [file pone.0282854.s001.pdf]

DT1	DT2	DT3	DT4	DT5	CP1	CP2	CP3	CP4
5	5	6	4	6	7	6	5	6
5	7	5	7	4	7	7	6	4
6	7	6	5	5	4	6	5	5
7	5	4	6	7	7	5	4	6
4	5	7	6	7	7	6	6	4
5	5	6	7	6	5	6	6	7
7	6	6	6	6	7	6	5	6
6	5	6	5	7	7	7	5	6
5	5	5	6	5	5	6	6	7
6	7	6	6	7	7	7	5	5
5	6	6	5	4	7	3	6	6
7	5	4	6	6	5	4	5	5
5	5	6	4	6	5	5	6	7
5	7	4	4	5	7	5	7	6
6	5	5	6	6	5	6	6	4
6	6	7	5	5	6	6	6	6
5	6	7	6	4	4	7	6	4
6	4	7	6	6	7	5	5	5
6	5	4	6	4	6	6	5	5
4	5	6	7	4	7	4	6	5
7	5	7	6	5	7	5	7	7
6	6	6	6	6	5	6	6	7
4	5	4	5	4	4	7	6	5
5	4	6	4	6	7	5	6	6
7	5	4	4	6	6	5	5	6
5	6	7	6	4	7	5	5	4
5	7	4	6	5	7	5	6	5
5	6	6	5	6	7	7	5	7
4	6	7	6	5	6	5	6	6
4	6	4	6	6	5	5	5	7
6	7	7	6	4	5	7	6	7
6	6	5	6	5	6	7	5	7
6	4	6	4	4	7	6	5	5
6	5	7	5	7	7	6	6	6
6	4	5	6	6	6	5	5	5
6	6	6	6	7	7	6	6	6
5	5	7	5	7	4	6	6	5
5	5	7	5	7	7	6	6	7
6	5	7	6	6	7	5	6	7
5	5	6	5	6	5	5	4	4
5	5	4	5	4	7	5	7	5
7	5	6	5	6	7	4	5	5
6	4	7	4	5	6	7	6	5
6	6	4	5	4	7	6	6	5
5	5	7	5	5	7	7	6	4
4	5	6	6	4	6	7	6	7
7	5	6	7	7	5	5	5	4
7	6	6	6	6	7	6	6	4
5	7	7	5	4	7	5	7	5
5	7	5	4	7	7	7	6	6
6	7	4	6	5	7	6	6	5
5	6	4	6	5	7	7	5	5
5	5	7	6	7	7	5	6	5
5	5	7	6	7	7	6	4	6
6	5	7	6	5	5	7	6	5
5	6	7	7	6	7	6	6	5
7	5	5	7	4	7	5	7	7

6	5	7	6	4	5	5	5	5
4	6	7	4	4	5	5	6	6
7	5	6	6	5	5	6	6	6
5	5	6	6	7	7	6	5	7
5	5	6	5	6	7	5	5	7
6	7	5	6	6	6	5	5	7
6	5	7	5	6	7	6	6	6
7	5	6	7	6	7	5	5	5
6	7	6	4	7	5	6	5	6
6	6	4	6	4	5	6	5	6
4	5	6	6	6	6	5	6	4
7	5	4	6	7	7	6	6	5
5	5	3	5	4	3	5	4	5
5	6	6	4	5	6	7	6	5
6	4	7	5	4	7	7	6	4
5	5	5	6	4	5	5	5	7
6	4	7	5	6	5	5	6	7
4	7	4	5	6	7	6	5	7
6	7	5	6	6	7	7	7	7
5	5	6	6	7	5	5	5	6
4	4	7	5	7	4	5	7	5
4	7	5	5	5	7	6	6	4
5	5	4	4	4	7	7	6	7
6	5	7	6	5	5	4	6	5
6	5	7	5	5	7	6	6	6
6	5	6	4	6	7	5	5	5
4	7	6	5	4	7	6	5	4
6	6	7	4	7	7	7	6	7
7	5	7	6	7	5	6	6	7
6	5	6	6	6	7	5	6	7
6	6	7	4	4	4	5	6	5
4	6	7	6	4	5	5	5	6
6	5	7	6	6	5	6	6	5
5	6	4	6	7	7	7	7	6
5	6	4	6	7	7	7	7	6
5	6	4	6	6	6	5	5	6
4	5	7	4	6	7	5	6	7
7	5	7	6	6	7	4	5	4
4	7	4	6	5	6	7	7	7
6	4	6	4	6	7	5	6	5
5	7	7	6	6	5	7	5	4
6	6	7	6	7	5	7	6	6
6	5	7	6	6	7	5	6	6
5	5	7	6	4	4	6	5	5
5	5	6	6	7	7	7	5	4
5	5	7	5	6	6	7	5	6
5	4	4	6	4	4	5	4	5
7	6	7	5	5	6	7	6	7
5	7	4	6	5	5	6	5	5
5	5	6	6	5	5	6	5	7
7	5	6	6	4	5	7	6	6
6	5	6	7	6	6	3	5	4
5	6	7	4	6	5	7	5	4
6	6	6	7	4	7	5	5	4
6	6	5	7	4	5	6	6	5
4	6	5	6	6	4	5	6	5
5	4	4	6	4	7	6	6	6
7	5	7	4	4	5	7	5	6

6	5	4	7	5	6	7	6	4
4	5	6	5	5	6	5	4	6
5	5	4	6	4	7	7	5	4
4	7	7	7	4	7	7	5	6
6	4	4	6	6	6	7	5	7
5	7	6	6	5	4	7	5	5
6	7	4	5	6	3	5	5	5
6	4	5	6	5	7	6	6	5
6	5	4	6	5	7	5	6	6
5	5	4	4	4	7	7	6	7
5	5	4	4	4	7	6	6	7
5	5	4	5	5	6	7	5	6
5	5	4	4	5	6	6	6	5
5	5	4	6	6	7	5	5	6
5	5	7	5	5	5	5	5	4
7	5	7	5	4	7	7	5	7
4	7	4	6	5	5	6	5	4
5	5	4	6	6	5	5	5	5
5	5	6	6	5	7	6	7	6
6	5	5	4	4	7	6	6	7
7	5	5	4	5	4	6	5	6
5	4	7	6	6	4	5	6	7
4	6	5	6	7	5	6	5	5
4	7	4	6	4	7	7	5	5
6	5	4	5	7	4	6	6	4
6	4	6	5	4	7	6	5	7
5	4	5	6	6	7	7	6	5
5	6	4	7	6	7	5	6	6
6	4	6	6	6	7	7	6	5
5	6	5	5	4	4	5	6	6
5	4	7	6	5	7	5	5	5
5	7	7	6	6	5	6	5	7
5	7	6	7	4	5	6	5	7
6	6	6	7	7	6	7	6	7
6	5	4	5	4	7	6	6	7
6	6	7	6	5	5	5	6	7
5	5	4	7	6	7	7	6	6
6	7	6	6	7	7	6	5	7
4	5	7	6	6	7	5	5	7
4	4	6	6	4	7	5	6	6
6	7	7	5	7	6	6	6	5
6	5	6	6	4	5	6	5	6
5	4	4	5	5	7	7	5	4
6	5	6	7	5	6	7	6	7
3	4	3	6	6	3	6	6	4
6	7	6	4	5	7	7	7	7
6	7	4	6	6	7	6	5	6
5	5	7	6	5	4	5	6	6
5	5	7	6	5	4	5	6	6
5	5	7	6	5	4	5	6	6
7	5	7	6	5	6	6	5	4
6	7	7	6	5	5	6	6	7
5	4	5	6	6	7	6	5	6
4	5	7	4	5	5	5	5	7
5	5	7	5	5	7	7	5	6
4	5	7	4	4	6	6	6	4
7	4	6	4	4	7	6	5	5
6	5	7	6	6	7	6	6	7

4	5	5	6	4	6	5	5	7
6	6	7	5	7	7	6	6	6
5	4	7	7	5	7	7	6	4
6	7	6	5	5	7	5	6	4
5	5	7	5	6	7	5	6	4
6	5	5	5	5	4	6	6	7
5	6	7	6	6	5	6	5	5
6	5	4	3	3	3	5	6	6
4	5	6	7	6	6	5	6	5
5	3	3	5	5	5	5	6	3
5	5	4	4	6	3	4	6	3
5	5	4	6	5	6	6	5	5
6	6	7	5	6	6	7	7	7
4	7	7	4	6	7	6	6	7
5	5	7	5	5	7	7	7	6
6	7	7	4	6	6	7	6	7
5	5	4	5	4	7	5	6	5
4	7	5	7	5	5	6	6	5
4	7	7	6	5	5	7	6	7
5	5	6	6	5	5	5	5	5
7	7	4	6	5	5	7	5	5
7	7	5	5	4	6	5	5	6
6	4	6	6	5	7	7	6	7
6	7	5	4	4	7	7	6	5
5	5	6	6	6	5	6	5	6
6	5	6	5	7	4	7	5	7
6	5	6	5	4	5	7	5	6
5	7	4	6	6	6	5	6	4
4	7	7	7	4	6	4	6	7
5	5	7	6	6	5	7	6	7
6	5	5	6	4	7	5	6	6
6	5	6	6	5	7	5	5	6
5	4	4	7	5	7	7	5	5
6	4	4	4	6	7	5	6	4
6	6	4	5	6	7	6	6	6
6	4	7	7	5	7	7	5	4
5	4	7	6	4	5	5	6	4
6	5	6	7	5	5	3	6	7
6	4	4	5	6	6	5	5	4
6	5	7	6	6	7	7	6	6
5	5	7	6	5	5	6	6	6
5	5	7	4	7	5	6	5	4
6	6	3	5	5	5	5	6	4
6	5	6	5	6	6	7	6	5
6	7	6	6	5	6	7	6	7
6	4	7	6	4	4	7	5	6
6	5	4	4	4	7	5	5	4
6	5	3	4	5	4	5	5	3
6	5	6	6	6	3	3	6	3
5	6	5	6	6	6	3	3	3
6	3	3	5	4	5	5	5	3
4	5	7	4	5	7	5	6	5
5	5	6	6	5	3	3	6	3
6	7	4	7	4	7	5	5	5
3	4	6	4	6	4	5	5	5
5	5	4	5	4	3	6	5	3
5	3	3	5	6	3	3	5	3
6	4	4	4	6	7	6	5	6

5	6	4	4	4	7	5	5	6
6	5	4	5	4	7	5	6	6
3	4	3	6	4	4	5	6	5
6	5	6	4	5	3	5	5	6
3	4	6	5	5	4	5	5	6
5	5	6	4	4	5	5	4	5
6	5	6	6	7	7	7	5	6
6	6	6	5	5	5	7	6	5
4	5	7	6	5	7	5	5	7
6	5	7	6	7	5	7	6	6
6	6	5	5	4	5	7	5	6
4	5	7	5	5	7	6	5	6
7	5	7	5	6	7	7	5	6
6	5	7	4	6	7	5	6	5
5	5	6	6	7	7	5	6	6
7	7	6	6	6	7	5	6	7
7	5	7	6	7	5	7	6	5
5	5	4	4	4	7	6	5	7
5	5	7	6	4	6	6	5	7
3	6	3	6	5	3	6	6	3
6	3	3	6	5	3	6	5	6
6	6	6	6	6	5	6	6	7
3	5	6	5	5	3	5	6	5
5	6	5	6	6	3	5	5	3
3	6	6	6	6	5	5	5	3
3	3	6	5	4	5	6	6	3
3	3	5	6	5	3	6	6	4
6	6	4	6	5	3	6	6	4
4	6	5	7	7	7	6	6	7
5	7	6	6	7	6	7	6	5
6	6	7	4	4	6	6	7	4
6	5	7	6	5	6	5	6	6
6	5	7	5	6	7	5	7	4
6	5	5	5	4	7	7	4	4
6	7	7	6	4	7	6	5	5
5	4	7	6	4	7	5	6	7
5	4	4	6	4	5	6	5	6
4	5	6	5	7	7	5	5	5
6	3	6	4	5	3	6	5	5
4	7	7	7	7	6	5	5	7
6	6	6	5	4	6	7	6	7
5	5	6	3	3	5	3	6	5
6	3	3	4	6	5	5	6	6
6	6	3	6	6	5	6	6	4
5	4	3	6	5	6	5	5	6
6	5	7	6	4	5	7	7	4
6	5	4	6	6	6	5	6	3
6	5	6	6	4	6	6	6	3
6	5	6	4	5	3	5	5	6
5	5	3	6	3	3	5	6	3
5	5	3	6	4	5	5	6	5
6	5	6	6	6	3	3	5	3
6	5	6	6	3	3	3	5	5
6	6	7	5	6	7	6	7	6
3	3	3	4	5	3	6	5	3
6	5	5	6	6	3	6	6	4
3	5	5	6	3	3	6	5	5
5	5	6	3	6	6	5	5	5

5	3	4	5	5	3	5	5	6
6	5	4	6	3	5	6	5	3
6	5	3	6	3	5	6	5	6
3	3	6	3	3	3	5	6	4
6	5	6	6	4	6	3	5	5
5	5	5	6	4	5	6	6	6
5	3	6	6	3	5	3	6	6
3	5	5	6	3	5	5	5	5
5	5	3	6	6	5	6	6	4
6	5	6	6	6	6	3	6	4
4	5	5	5	6	2	6	6	5
7	5	7	6	5	7	6	5	4
3	4	3	4	3	5	6	5	3
7	5	5	4	7	6	5	5	5
7	5	5	4	7	6	5	5	5
3	5	6	6	6	7	5	5	6
6	5	7	5	4	5	7	6	6
6	3	6	6	3	3	3	6	5
6	5	4	5	5	6	5	4	3
3	5	4	4	4	5	5	5	6
6	3	6	3	5	3	6	5	6
6	6	4	6	5	5	6	6	6
3	5	6	4	6	5	3	5	4
5	5	6	5	6	3	5	6	6
5	5	6	5	4	3	6	5	5
5	5	3	4	5	3	5	5	3
6	5	3	6	5	3	6	6	6
6	5	3	5	4	4	5	5	5
3	5	4	6	5	6	6	6	4
6	5	3	3	5	3	6	5	6
4	3	3	5	5	4	3	5	5
6	6	4	5	3	3	5	6	4
6	3	3	6	6	3	5	6	5
5	5	3	4	4	3	3	6	6
5	4	3	4	6	3	3	5	4
5	3	3	6	3	5	6	6	6
3	5	3	6	6	5	3	5	6
3	6	3	5	5	3	5	5	4
5	5	3	5	4	6	3	5	6
6	5	6	6	5	3	5	5	6
4	5	6	4	6	3	5	5	5
5	5	5	4	4	4	4	5	6
6	5	3	6	4	3	3	5	6
5	5	6	3	6	5	6	5	5
4	3	3	5	6	3	5	5	3
6	5	5	7	5	7	6	6	4
6	3	6	6	6	3	3	5	6
3	3	3	6	4	3	5	5	5
6	4	4	6	5	4	5	7	7
5	3	4	4	5	5	3	5	6
5	5	5	5	5	5	6	6	3
5	6	3	6	4	3	3	5	5
6	6	6	6	5	3	3	5	5
5	5	6	6	3	5	3	5	5
6	5	6	5	6	3	5	6	3
6	3	6	5	5	3	3	6	5
6	4	5	6	6	3	5	5	6
5	5	6	6	6	3	6	6	6

6	5	3	6	3	4	5	4	5
3	5	6	6	4	5	6	6	3
6	5	6	6	6	5	3	5	3
6	5	3	5	6	3	3	5	6
6	5	6	6	3	5	5	6	6
5	3	6	3	6	5	6	6	3
5	5	5	4	5	3	3	6	6
6	3	5	3	3	3	5	4	3
5	5	5	6	6	3	3	6	5
6	5	6	6	5	3	6	6	6
5	5	6	5	6	5	5	5	5
5	4	5	5	5	3	3	6	3
5	3	3	3	5	3	5	6	6
6	4	5	5	5	5	6	6	5
3	3	3	6	4	3	5	5	6
6	5	3	6	5	3	5	6	6
5	5	5	5	5	3	3	5	3
5	5	3	6	5	3	6	6	6
4	5	7	7	7	4	5	5	4
5	3	5	6	5	3	3	5	4
4	7	4	7	4	7	6	4	6
6	5	6	3	4	3	3	6	3
5	3	6	3	4	6	3	6	6
5	5	3	4	6	6	6	5	5
6	3	3	5	3	3	3	5	5
6	5	5	6	6	5	3	5	3
5	5	6	3	3	5	6	5	3
6	5	6	5	5	6	3	5	6
6	3	5	5	6	3	6	5	6
6	4	6	6	6	5	5	5	5
3	6	5	6	4	3	5	5	5
6	4	4	6	6	3	5	5	3
5	5	2	5	4	5	3	2	3
6	3	5	4	5	6	5	6	6
6	5	3	6	6	3	6	5	6
6	3	3	6	4	4	5	5	6
6	4	6	5	3	6	3	5	3
6	5	3	6	5	3	5	6	5
6	3	3	6	5	5	6	5	3
5	5	4	3	3	3	5	5	5
5	6	3	3	6	6	6	6	5
6	5	6	6	4	3	5	5	3
5	6	6	6	3	5	3	6	5
6	5	5	5	3	5	5	6	3
3	3	5	5	4	5	2	2	2
5	6	6	5	3	3	5	6	6
3	3	6	5	6	3	6	5	6
5	4	3	4	6	3	5	5	5
5	3	3	6	4	3	4	5	5
5	5	3	4	5	6	5	6	3
6	5	5	6	3	3	6	5	5
3	3	5	6	5	3	3	5	6
6	3	6	4	3	3	5	5	4
5	5	3	6	6	3	5	5	3
5	5	3	4	5	5	3	5	5
5	5	6	4	6	6	5	6	3
5	5	3	6	6	5	6	6	6
5	3	3	6	6	4	6	6	3

5	4	5	6	5	3	6	6	5
5	5	5	6	3	5	3	5	6
2	5	5	2	2	3	3	2	3
5	3	5	2	5	3	5	5	5
5	5	6	6	6	6	5	6	3
6	5	4	3	4	6	6	6	5
6	3	3	3	6	4	3	6	3
3	5	5	6	3	5	3	5	5
5	5	3	5	6	3	5	6	3
3	6	4	6	6	3	3	6	6
4	5	3	6	3	5	5	5	5
6	3	4	3	6	4	5	5	6
5	5	3	6	6	5	5	5	6
5	5	3	6	3	3	6	6	5
3	3	5	2	2	5	2	2	4
3	5	2	4	5	3	2	5	3
3	5	3	2	3	5	3	5	5
5	5	4	2	4	5	2	5	2
3	3	3	5	4	5	5	3	3
6	5	3	4	6	5	5	6	4
6	5	3	6	3	3	5	5	6
5	5	3	3	5	5	5	6	6
5	5	2	2	5	5	3	2	5
2	5	5	4	5	5	2	5	4
3	5	4	6	6	3	5	5	3
5	5	6	5	4	5	3	6	6
6	5	3	3	3	5	5	5	3
5	6	6	3	4	5	5	6	3
5	5	5	6	3	3	5	6	5
5	5	6	5	6	5	3	6	4
2	5	2	2	3	3	3	2	2
6	5	4	6	5	3	5	5	6
5	5	3	3	4	5	3	5	5
5	2	5	4	2	3	2	5	3
2	5	3	2	2	3	5	5	2
2	3	3	5	5	3	3	5	5
3	2	3	2	4	3	5	5	2
2	5	3	5	3	3	3	5	5
5	5	3	5	4	4	5	2	5
2	5	3	5	2	5	2	2	5
2	5	2	2	5	3	5	2	3
2	5	5	2	2	5	5	2	4
2	5	2	2	3	3	5	2	5
5	3	3	2	4	5	3	2	3
2	3	5	5	5	3	2	2	3
2	5	5	5	4	3	3	2	5
3	5	2	5	5	5	3	5	4
5	5	5	2	5	5	5	5	5
5	3	4	5	2	5	2	5	5
6	6	3	6	3	5	5	5	3
5	5	3	5	2	3	2	4	4
2	2	5	2	2	3	5	2	2
3	5	2	2	2	2	2	2	2
2	5	3	5	5	2	2	2	2
5	5	2	5	2	2	2	2	2
2	5	3	5	2	3	2	2	3
3	5	4	5	5	3	3	5	5
5	2	3	2	3	3	5	5	3

3	4	3	2	5	5	5	2	2
3	5	3	4	5	5	2	5	5
2	5	3	2	3	2	3	2	5
3	5	3	3	5	3	5	5	5
3	5	4	2	2	3	2	5	2
5	5	3	2	5	3	5	2	3
3	5	3	2	3	5	3	5	5
2	2	5	5	4	5	3	5	5
4	2	4	5	5	3	3	2	3
2	3	2	5	5	3	5	2	3
5	5	5	4	5	3	3	2	2
2	5	3	3	5	2	2	5	2
2	3	3	5	3	5	3	5	5
2	2	2	5	2	3	2	2	3
3	5	4	5	4	2	2	2	5
2	3	3	3	2	3	2	5	3
5	5	2	5	3	3	3	5	3
5	2	3	5	5	3	5	3	5
5	5	4	5	2	5	5	2	4
2	3	2	5	2	3	2	5	2
5	5	3	5	5	4	2	5	3
5	5	5	2	3	3	2	2	3
2	5	5	4	2	3	2	2	5
2	3	3	5	2	2	5	5	3
5	3	3	5	5	5	2	5	5
5	5	3	3	5	3	5	2	2
2	2	2	2	3	3	3	5	2
5	2	5	3	2	3	2	5	5
2	5	3	2	3	3	5	2	3
2	5	4	5	5	3	3	5	3
5	5	2	2	5	5	5	5	2
2	5	3	5	3	3	5	5	2
2	5	3	5	4	5	5	2	5
2	5	4	5	2	3	2	5	3
5	5	3	2	2	3	2	2	3
5	5	3	2	5	5	5	4	4
5	3	4	5	5	3	5	5	3
2	5	5	5	4	5	5	5	3
5	3	5	4	2	2	5	5	5
5	5	2	2	2	5	5	2	2
5	5	4	5	5	3	5	5	4
2	5	2	2	4	2	2	2	5
5	2	2	2	5	3	5	2	2
5	5	3	3	5	5	5	2	5
1	1	3	1	1	1	2	2	4
1	3	2	2	4	3	3	1	1
5	5	2	2	5	3	2	5	5
1	1	2	2	1	2	1	1	2
1	1	2	2	2	1	1	1	3
1	1	3	4	1	1	1	1	4
1	1	4	2	1	3	1	2	2
2	1	1	1	4	1	2	1	3
2	4	2	2	2	3	1	1	2
3	1	3	2	3	3	2	2	2
2	1	2	3	1	3	2	1	3
5	5	4	4	3	3	3	2	5
5	5	3	5	3	3	2	5	2
1	1	4	1	2	3	2	1	3

2	3	2	5	5	3	2	2	3
2	1	3	1	1	2	2	1	2
2	1	2	2	3	2	1	2	1
1	3	2	1	1	2	2	1	2
1	1	3	1	1	3	1	1	2
4	1	3	2	2	1	1	2	1
2	1	3	1	1	3	2	1	2
3	2	2	1	4	3	1	2	2
1	1	1	1	3	2	1	2	3
1	1	4	1	2	1	1	3	2
3	1	4	1	1	1	2	1	1
2	1	1	3	3	1	3	2	3
1	1	3	4	1	1	1	2	3
2	4	3	2	2	3	1	1	2
2	4	2	2	1	3	3	1	1
3	1	3	3	1	3	1	2	1
1	1	2	1	3	3	2	3	3
2	1	2	2	1	1	1	2	2
1	1	3	2	4	3	3	2	1
2	4	4	1	2	2	1	2	1
1	1	3	1	3	1	1	1	4
2	1	4	3	1	1	3	2	4
2	1	4	3	1	1	3	2	1

CP5	FP1	FP2	FP3	FP4	Geo	Staff	Year	Sales
6	6	5	7	4	3	1	4	4
4	5	7	7	7	2	4	2	1
4	6	7	5	5	1	1	4	2
6	6	7	6	4	3	4	4	3
6	6	6	7	6	1	4	2	4
6	7	6	6	4	4	3	3	1
7	5	7	5	7	1	3	6	1
5	7	5	5	7	4	3	5	1
5	7	7	7	4	3	4	3	1
4	6	6	5	7	6	4	2	7
6	6	7	6	4	3	1	2	2
6	6	7	5	4	3	2	2	1
5	5	5	6	7	5	3	4	3
7	7	6	6	7	3	2	3	6
4	7	5	5	7	3	1	3	3
6	6	7	6	7	1	2	2	5
7	5	5	6	7	3	1	4	2
7	6	7	5	7	4	3	4	6
6	6	7	6	4	3	1	3	4
4	6	6	7	4	4	3	2	2
4	7	5	5	6	4	2	2	1
6	6	5	6	4	4	2	3	1
7	7	7	7	7	5	2	4	2
7	5	5	7	7	1	1	3	6
7	6	7	7	7	3	1	3	1
7	5	7	7	7	3	2	6	6
5	7	5	5	7	3	2	3	2
6	6	5	6	6	1	2	4	1
6	7	5	7	4	4	4	3	3
6	7	5	6	7	4	4	6	3
7	6	6	7	4	2	4	3	3
6	6	5	5	4	3	2	3	5
4	6	7	7	7	4	3	3	7
6	7	5	6	4	2	2	4	5
6	5	7	7	6	4	3	3	6
7	5	5	7	7	1	2	3	3
5	7	6	7	7	4	3	2	2
6	6	6	6	6	4	3	2	4
4	6	6	7	5	2	1	4	7
6	7	5	6	4	2	2	2	2
6	7	6	6	7	1	4	3	2
4	7	7	6	6	2	3	4	6
5	7	7	7	4	5	2	2	1
5	5	5	5	4	7	1	2	3
7	6	5	7	4	3	3	2	1
5	7	7	7	4	6	3	4	1
6	7	7	6	4	6	2	2	6
7	7	6	7	5	2	2	4	1
7	4	6	6	7	6	3	3	4
5	6	6	6	6	2	2	3	3
4	4	6	6	7	5	3	2	1
7	5	7	7	6	4	2	3	7
5	6	5	7	7	3	3	3	2
7	5	4	6	6	5	2	3	2
4	6	6	6	6	4	2	4	5
7	5	5	7	7	1	1	2	1
7	6	7	7	7	2	2	4	2

6	7	5	6	4	1	2	2	2
7	6	5	7	4	1	4	2	5
5	7	6	5	7	3	3	4	4
5	6	6	7	6	1	3	3	3
7	7	7	5	6	2	2	3	1
7	6	7	7	7	2	3	6	1
6	7	5	7	4	5	2	3	1
7	6	6	7	6	3	2	6	7
7	7	5	5	7	4	2	4	2
4	6	7	7	6	3	3	4	2
4	6	6	5	6	6	4	3	6
5	6	7	4	4	7	3	3	1
6	5	3	3	3	5	2	3	5
7	5	5	6	6	1	2	6	2
6	5	6	6	4	3	4	2	1
6	7	7	6	4	1	3	2	2
7	5	6	6	5	3	3	4	3
7	6	5	7	4	4	4	6	3
7	5	6	7	6	5	2	3	3
7	7	6	7	6	5	2	3	3
6	7	7	7	6	1	2	2	1
6	6	7	6	5	3	3	6	3
6	7	5	7	7	4	2	2	2
7	5	7	5	4	1	2	3	1
5	5	5	6	4	5	3	6	4
7	6	6	6	6	1	1	6	2
7	6	6	6	6	5	4	4	6
4	6	6	5	7	3	2	4	6
5	5	7	7	7	4	2	4	5
7	4	5	6	6	6	3	2	3
7	7	4	7	7	1	4	4	5
7	5	5	5	4	5	2	3	7
7	5	6	7	7	3	3	2	1
6	5	5	6	7	2	3	2	4
6	5	5	6	7	2	3	2	4
6	7	7	7	7	7	3	2	3
6	6	5	5	7	6	2	5	1
7	5	7	5	4	1	3	3	1
6	7	7	7	7	1	2	2	4
6	7	7	6	7	3	3	3	4
6	5	5	5	6	4	3	6	5
7	6	6	7	7	4	2	2	6
5	4	5	6	4	2	2	4	1
5	5	6	7	4	4	2	3	2
7	6	6	6	6	2	4	3	3
7	6	5	6	4	3	3	4	3
5	5	6	6	6	4	4	2	1
7	6	5	6	6	2	2	3	5
6	7	5	6	6	2	3	3	3
5	6	7	7	6	4	2	3	4
5	5	5	7	7	2	3	3	1
6	7	6	7	4	2	2	4	4
6	5	5	7	5	7	4	3	2
7	6	6	7	7	6	3	3	4
6	5	7	6	6	1	2	3	7
6	5	7	4	4	1	4	3	4
6	5	5	7	7	6	2	3	1
4	5	6	7	6	5	4	3	2

6	5	6	7	4	4	4	4	2
7	6	5	7	7	4	2	6	3
7	5	6	5	4	3	4	4	4
7	6	5	5	7	2	4	2	2
7	5	7	7	4	2	2	4	5
6	5	7	7	6	1	3	2	1
7	6	7	7	7	3	4	4	4
6	7	5	7	4	4	2	2	5
7	5	5	6	6	3	4	3	1
5	6	7	5	4	4	2	4	4
5	5	5	7	6	5	4	3	1
6	6	5	7	4	3	3	3	1
4	4	6	7	6	1	4	2	6
7	5	5	6	6	7	2	2	1
5	5	5	7	4	4	1	6	4
5	7	5	5	7	5	2	3	2
6	6	7	7	6	2	1	2	2
5	4	5	7	5	4	3	2	2
6	5	6	6	7	1	2	6	2
5	6	6	7	4	1	3	3	2
5	5	5	7	6	2	3	4	2
6	6	7	7	4	2	1	6	6
7	7	6	6	5	4	2	4	2
6	6	7	6	7	5	4	2	3
6	5	5	7	6	4	2	4	7
5	6	5	6	4	6	4	4	7
5	6	7	7	4	1	2	2	2
6	7	5	7	4	4	2	4	3
4	6	5	7	7	4	3	3	2
4	7	7	5	6	6	4	2	1
7	7	5	7	6	3	3	2	2
4	4	5	6	4	1	3	6	3
6	5	6	7	7	1	3	4	2
6	6	5	4	7	4	2	2	2
6	5	6	7	6	4	1	4	5
5	6	6	7	4	4	3	3	4
6	6	6	6	7	3	1	3	1
6	6	7	6	7	2	3	3	2
5	6	5	7	5	3	3	6	3
6	7	5	7	7	3	2	2	1
7	7	5	7	6	6	3	3	1
7	6	5	7	4	5	3	3	5
7	5	6	6	7	2	2	2	1
7	5	6	5	4	2	1	3	1
5	3	5	3	1	1	2	3	5
7	4	5	7	4	5	3	2	2
7	6	6	7	7	2	4	2	3
7	7	7	7	4	1	3	3	4
7	7	7	7	4	1	3	3	4
7	7	7	7	4	1	3	3	4
6	5	7	6	4	3	2	3	4
6	7	6	7	4	4	3	3	4
6	5	6	6	4	2	2	3	2
6	5	7	7	5	6	4	3	4
6	6	6	7	7	4	2	4	4
4	4	5	7	4	4	2	3	7
7	7	7	7	6	4	2	2	4
6	6	5	6	4	1	2	3	5

7	7	7	5	6	1	2	2	1
4	5	7	5	6	1	3	2	2
6	7	7	5	6	1	2	4	2
7	7	7	6	7	6	4	4	5
7	6	7	5	6	1	3	3	1
7	5	5	6	5	1	2	3	1
7	5	7	7	4	4	2	2	1
3	5	6	3	5	4	4	2	1
6	7	6	7	4	2	2	3	6
3	3	5	3	6	1	3	6	1
3	5	5	5	4	4	1	6	3
7	6	6	6	7	3	4	6	1
6	6	6	6	6	5	3	4	6
4	7	5	6	6	7	2	6	5
5	5	5	5	4	7	3	3	1
6	7	5	7	7	5	1	2	5
5	6	7	6	7	4	4	4	2
6	6	7	5	5	4	2	3	3
7	6	5	6	4	2	4	3	7
7	7	5	6	4	5	2	5	5
7	7	5	6	5	4	4	6	2
7	7	5	6	6	1	3	3	7
7	5	5	6	7	2	2	3	2
4	7	6	7	6	2	2	2	2
4	6	5	5	4	2	2	3	2
7	7	6	6	6	5	4	6	1
6	7	6	5	4	4	3	3	6
7	7	6	6	7	1	2	3	3
5	5	7	7	4	1	4	2	3
5	6	7	7	5	2	3	2	3
6	6	6	6	4	1	3	4	2
4	5	7	7	4	1	3	3	1
4	5	6	4	6	4	1	4	3
6	7	5	5	4	2	4	2	2
5	7	5	7	7	4	4	3	1
5	7	6	6	7	3	4	2	6
6	7	5	6	7	4	2	4	6
7	6	5	5	7	2	2	3	6
7	5	6	5	7	3	2	6	1
7	5	5	7	6	1	3	3	3
4	5	7	6	7	2	2	2	7
7	7	6	7	6	1	3	3	3
4	3	3	5	6	2	1	6	2
5	4	5	6	7	4	2	2	5
6	6	5	6	4	2	3	4	2
6	5	5	6	7	2	3	3	1
4	6	6	7	7	3	1	2	3
6	3	5	3	4	2	3	2	1
5	6	5	3	3	4	3	3	2
6	6	3	6	6	4	1	3	3
6	3	5	3	1	4	4	3	1
4	5	6	5	4	2	2	3	5
6	6	3	6	1	3	3	3	2
7	5	6	6	6	7	2	3	3
6	3	5	6	1	3	2	3	2
3	3	3	5	3	6	3	2	6
5	5	5	6	6	3	3	3	1
7	7	5	7	7	4	2	3	1

4	7	6	7	7	1	4	4	5
6	7	7	5	6	6	3	3	3
5	5	3	6	3	6	3	3	2
5	6	3	6	1	4	2	3	5
5	6	5	6	3	1	2	6	2
5	5	5	3	6	2	3	2	5
4	6	6	7	4	4	3	3	2
7	6	7	7	6	7	2	4	7
6	6	7	5	4	1	3	4	1
5	5	7	7	7	5	3	6	3
5	5	5	4	6	2	3	3	3
5	7	6	5	4	3	4	3	7
5	5	6	6	6	1	4	3	3
6	5	5	7	6	6	3	3	4
7	5	7	7	4	3	4	3	1
7	7	5	5	5	7	3	3	3
6	6	5	7	5	2	2	3	4
7	5	5	6	7	1	3	3	6
5	7	5	6	5	7	2	2	3
6	5	6	6	6	4	2	4	1
4	5	3	6	6	3	4	2	2
7	7	5	7	7	5	2	4	3
4	4	6	6	1	6	3	3	1
6	4	6	6	5	3	2	2	7
6	6	5	5	6	2	3	6	6
3	6	5	3	6	1	2	4	4
3	5	5	6	6	1	3	4	3
4	6	3	6	1	2	2	4	1
6	5	6	7	6	1	2	2	1
5	7	5	5	7	2	2	4	1
6	7	7	6	7	1	3	2	2
4	6	6	7	7	2	3	2	1
7	5	5	4	4	1	3	4	2
5	7	6	6	6	1	2	2	3
6	5	4	7	6	2	3	3	1
5	5	6	7	4	4	1	4	3
4	7	5	4	5	1	3	2	4
5	7	5	7	6	1	3	4	6
3	5	6	6	6	1	4	6	3
7	4	7	6	6	1	3	4	2
6	7	7	6	6	2	4	3	4
3	3	3	5	6	3	2	4	6
3	6	3	6	3	6	4	3	6
3	3	5	3	1	6	3	4	1
6	5	5	3	6	3	2	2	1
6	7	6	5	7	3	3	3	2
5	6	5	6	6	4	3	4	1
3	3	3	5	5	3	4	2	2
6	4	5	5	4	2	3	4	3
3	6	6	3	3	3	4	3	3
4	3	6	3	6	1	4	6	7
4	5	5	4	1	6	3	4	4
3	5	6	3	3	6	3	3	6
6	7	6	7	7	6	2	2	5
4	5	5	3	4	4	1	3	1
3	6	6	3	3	6	3	6	1
6	6	3	3	6	4	2	3	5
6	6	6	3	6	1	3	2	3

4	5	5	6	6	4	2	4	1
5	5	5	5	3	3	3	3	1
6	5	5	5	6	2	4	4	7
6	5	6	3	6	1	3	3	5
6	3	3	6	6	1	4	5	7
4	6	5	3	3	4	3	3	4
4	5	5	6	3	4	2	4	7
6	3	5	5	3	3	4	3	6
5	5	6	3	6	2	2	2	2
5	6	5	3	6	7	4	2	3
7	7	7	7	7	4	3	2	4
7	7	5	7	7	6	4	3	2
3	6	5	3	4	2	3	4	2
6	7	6	7	6	2	3	2	1
6	7	6	7	6	2	3	2	1
6	5	5	7	6	1	2	2	4
4	5	6	6	6	5	3	4	1
6	5	5	3	4	2	2	2	1
3	6	5	3	6	4	4	4	2
6	5	5	3	3	1	2	6	4
6	3	6	3	3	4	3	3	1
3	3	6	6	3	3	4	2	3
6	6	5	3	3	3	3	4	4
5	6	3	5	3	2	2	3	6
3	3	5	3	4	3	4	4	1
3	6	6	4	3	3	3	6	2
5	6	5	3	5	4	1	2	4
6	3	5	5	1	1	1	5	3
6	6	5	3	5	2	2	4	5
6	3	3	5	1	7	3	4	4
4	6	3	6	3	3	2	4	1
6	5	3	5	5	4	3	2	1
3	3	5	3	6	1	3	6	1
3	5	6	3	6	7	2	4	2
3	5	5	6	6	1	2	3	4
3	3	3	6	1	5	1	4	1
6	5	5	6	6	7	2	6	2
6	3	3	3	6	1	4	3	1
4	3	6	5	3	4	1	2	6
6	6	6	6	3	1	2	3	3
5	5	5	3	5	4	4	2	6
5	5	6	3	6	6	3	6	2
6	6	6	6	5	4	3	3	4
4	6	6	3	3	3	2	3	2
5	5	5	6	3	7	3	4	1
6	5	5	5	6	4	2	4	6
6	6	5	3	3	1	4	3	1
6	5	5	3	1	4	3	3	6
6	6	5	7	7	4	3	2	1
5	6	6	6	3	2	3	2	5
5	5	6	5	1	6	2	3	1
3	6	6	5	1	2	1	3	4
6	6	3	5	6	2	2	5	1
3	3	5	3	3	4	3	2	3
4	5	5	6	6	1	2	3	1
4	3	6	3	3	7	4	3	2
5	5	5	5	3	2	3	3	2
3	6	3	3	3	6	3	4	2

5	3	6	5	6	7	3	2	4
5	5	6	5	4	1	2	3	6
3	6	3	5	3	5	4	4	2
3	5	5	6	3	7	4	2	4
6	3	5	6	5	4	2	6	4
5	5	6	6	3	4	4	3	5
6	6	6	3	6	1	3	2	7
3	5	5	6	1	2	4	3	7
3	3	5	3	6	4	4	4	1
3	5	3	6	3	4	2	4	3
5	5	5	6	3	3	2	6	6
6	5	6	5	4	2	3	6	3
6	6	3	5	3	3	2	2	5
3	3	5	3	3	7	3	3	5
5	3	6	3	1	1	2	2	2
6	5	3	3	3	3	3	2	2
5	6	6	6	5	7	3	6	2
5	3	5	3	1	6	4	2	2
4	6	5	7	7	1	3	4	2
5	6	6	6	3	2	4	2	1
7	7	6	7	6	4	2	3	4
3	6	6	6	6	4	2	3	5
4	6	5	6	4	2	4	2	1
5	6	5	4	6	6	4	2	1
5	6	5	3	3	5	1	3	2
6	3	5	6	5	2	2	6	1
5	6	6	3	1	7	2	2	3
4	6	6	3	6	3	4	2	2
5	5	6	3	6	2	3	6	4
5	5	5	6	1	2	2	4	4
6	6	5	6	6	5	1	2	4
5	3	6	3	1	4	3	4	1
4	3	5	5	3	7	3	3	1
3	3	6	6	3	3	3	3	3
4	5	5	3	6	5	3	2	1
3	3	5	6	6	2	3	3	3
6	3	6	3	1	1	2	3	6
5	5	6	4	4	2	4	2	1
3	5	6	3	3	3	4	3	4
5	3	5	3	4	4	2	6	4
5	5	4	6	3	3	1	4	5
5	3	5	3	3	4	2	3	6
3	6	5	3	3	7	2	3	6
6	6	5	3	1	5	2	2	3
3	5	2	2	2	3	2	2	3
3	4	3	3	3	1	3	4	3
3	6	5	6	6	7	2	3	1
6	6	5	3	1	3	1	4	6
6	6	5	6	5	4	3	3	4
5	3	6	5	6	5	4	4	2
5	5	5	6	1	4	4	2	2
3	5	5	3	1	3	2	4	7
3	5	6	6	5	1	3	2	7
4	5	6	6	6	1	2	4	3
6	3	3	6	5	1	2	3	3
3	3	3	3	6	4	3	2	4
5	5	3	3	1	6	3	3	4
4	6	5	3	5	3	3	3	1

5	5	3	3	4	3	4	3	1
5	6	5	6	3	3	3	3	5
2	2	3	2	3	3	2	4	5
4	4	5	3	2	4	1	4	2
6	3	5	3	4	6	2	4	4
5	6	3	6	3	4	3	3	1
5	5	5	5	5	4	4	4	3
5	5	5	6	1	1	3	6	6
5	5	6	6	3	3	4	4	7
5	5	5	3	3	1	3	3	5
3	6	6	6	1	3	3	2	1
3	3	5	3	1	2	2	4	5
5	3	6	3	1	3	3	2	1
3	5	3	6	5	3	1	4	4
2	2	5	2	5	7	3	2	3
5	2	4	2	4	2	3	2	3
2	5	3	5	4	2	2	6	3
2	5	3	5	4	5	2	3	1
5	5	5	3	3	4	2	2	1
3	6	5	3	6	6	3	4	3
6	6	6	3	6	1	3	4	2
6	6	5	6	4	7	2	3	3
2	5	2	2	2	3	3	4	5
5	5	5	2	3	2	2	2	5
6	5	5	5	3	2	3	3	2
3	5	5	3	4	2	2	3	2
5	5	5	5	3	2	2	3	1
5	3	5	6	3	7	2	3	1
5	6	3	3	3	4	3	3	1
6	6	6	6	6	2	2	3	3
5	4	2	2	4	3	2	2	6
3	6	5	5	3	6	4	3	2
4	3	5	3	1	3	2	4	7
3	2	2	3	3	4	3	4	4
5	5	5	2	3	6	2	4	6
3	3	4	2	4	1	2	2	4
3	5	2	3	5	2	2	3	2
4	2	2	2	2	3	3	2	6
5	2	5	3	2	2	3	2	6
5	3	5	2	3	6	4	3	6
4	5	3	2	3	2	2	4	2
5	5	5	2	5	5	2	4	3
2	5	5	2	4	4	2	3	1
5	2	2	3	3	1	4	3	1
2	2	5	2	3	1	2	2	3
4	3	2	2	2	4	3	4	6
3	5	5	2	2	7	4	2	2
3	2	4	3	2	1	2	3	1
5	5	2	3	3	3	2	3	4
3	5	5	3	6	3	3	5	1
3	5	5	5	2	6	4	3	6
5	5	5	5	4	2	3	2	2
2	2	5	2	5	4	1	4	3
5	5	3	2	2	4	4	4	1
5	2	2	2	2	4	2	4	5
5	5	5	5	3	6	1	4	2
3	5	5	3	2	3	4	6	1
2	3	5	2	4	6	2	3	7

5	5	5	3	2	4	3	3	3
3	5	5	2	2	2	2	3	3
5	2	2	3	5	1	2	4	2
4	2	3	2	3	4	4	4	1
3	3	5	3	1	6	2	6	3
3	2	3	5	5	5	2	3	3
5	3	3	3	3	3	4	6	2
2	5	5	2	1	7	2	2	4
5	5	5	3	2	2	3	4	2
5	5	3	5	3	2	2	4	5
3	5	2	5	1	1	2	3	2
2	5	5	5	2	3	3	2	2
5	5	3	3	4	2	2	4	2
2	5	5	3	2	3	3	2	3
5	2	5	2	2	1	4	2	2
5	5	5	3	1	3	4	4	4
5	5	2	2	2	1	1	4	3
5	3	5	2	5	2	1	3	4
2	5	2	3	4	3	2	3	1
3	5	3	4	4	3	3	2	2
2	2	5	2	3	5	2	2	2
5	2	5	2	3	3	2	2	2
3	5	2	3	4	3	3	3	2
5	5	2	3	3	3	2	2	2
5	3	2	3	2	1	1	2	1
2	2	3	3	4	2	3	6	3
3	2	2	3	4	4	2	3	3
2	2	2	2	4	3	2	6	5
3	5	3	2	4	3	2	4	2
2	5	3	5	3	4	3	6	1
4	5	3	5	1	2	4	3	3
4	3	5	3	2	1	3	3	1
5	3	3	3	5	1	2	6	3
2	2	2	2	1	1	2	3	1
5	5	5	2	2	3	2	3	4
5	2	5	5	3	2	2	2	3
2	3	5	2	3	2	3	4	5
2	5	3	2	1	1	4	4	1
2	2	2	5	3	3	3	2	2
5	3	5	3	3	3	2	2	1
2	5	5	3	5	3	3	2	2
3	5	5	3	3	7	2	2	6
3	3	5	3	2	2	3	3	1
3	2	2	3	5	4	2	3	4
4	3	1	2	2	1	2	2	4
3	3	1	3	2	5	4	6	1
2	3	5	3	4	1	2	6	1
2	1	2	3	2	3	4	2	3
1	3	2	2	2	6	2	4	3
3	1	2	3	3	2	3	3	3
4	3	1	1	3	3	3	4	1
3	1	1	1	2	2	3	5	3
1	3	1	3	1	1	2	4	6
2	3	1	1	3	4	1	2	5
3	2	2	3	1	2	2	3	3
3	2	4	5	5	5	3	3	2
3	5	5	5	3	2	2	4	2
1	1	1	2	2	7	1	5	7

3	2	5	2	2	1	2	2	1
1	2	2	2	2	1	2	2	2
1	3	1	2	2	4	2	2	1
1	3	2	3	3	5	3	6	7
2	3	3	2	2	3	2	3	5
1	3	1	2	1	4	3	3	1
1	2	2	3	2	7	3	6	3
3	1	3	1	3	4	1	3	1
3	2	2	3	2	1	2	3	2
1	1	2	1	3	2	3	5	2
2	1	1	2	1	5	2	3	3
1	2	1	1	4	1	2	3	3
4	2	3	3	4	2	4	4	1
1	3	2	2	3	1	4	2	3
2	1	3	3	1	5	3	5	6
4	2	3	2	3	1	1	3	1
4	1	3	2	2	3	2	2	2
3	1	2	2	1	1	2	3	1
3	2	1	2	1	4	2	3	4
4	2	2	1	2	4	2	4	5
2	2	1	3	3	3	3	3	7
4	2	3	3	2	6	3	2	3
3	3	1	3	3	2	3	5	3

Industry	Random
8	0.339291
14	0.719758
10	0.046415
8	0.685039
9	0.82593
8	0.848519
9	0.600991
7	0.343694
3	0.947494
9	0.954442
8	0.366481
8	0.281364
3	0.119809
4	0.19913
14	0.446422
1	0.821023
4	0.564247
3	0.764815
9	0.890802
3	0.338121
5	0.216927
8	0.037874
5	0.369
10	0.548847
14	0.96943
4	0.638821
6	0.163371
2	0.45636
5	0.888006
14	0.794239
6	0.250445
14	0.786809
4	0.250334
8	0.018171
8	0.823182
2	0.31598
2	0.910934
6	0.094417
10	0.826586
8	0.356055
10	0.968758
3	0.159139
2	0.853137
10	0.934395
5	0.322275
4	0.200722
6	0.264423
7	0.5235
8	0.33973
7	0.827288
1	0.885069
3	0.737889
4	0.265664
5	0.443932
8	0.028703
1	0.392987
5	0.086032

3	0.087761
10	0.967586
3	0.530039
2	0.146618
3	0.941966
1	0.954918
1	0.364713
1	0.621952
4	0.46675
10	0.226618
7	0.01032
8	0.6907
4	0.419483
3	0.189874
7	0.87781
10	0.534445
5	0.040278
7	0.503224
4	0.405898
5	0.372834
9	0.272201
5	0.821802
1	0.99505
10	0.964797
5	0.845351
9	0.961513
10	0.232698
14	0.200728
6	0.893721
3	0.964263
2	0.030731
14	0.824227
6	0.780736
4	0.632263
4	0.800488
7	0.517421
5	0.99122
3	0.189193
1	0.260966
9	0.864863
8	0.110623
5	0.608581
6	0.615935
9	0.522075
10	0.385286
10	0.986969
5	0.361698
4	0.502304
4	0.753338
6	0.405459
6	0.156578
5	0.0855
3	0.102856
4	0.229921
3	0.983597
1	0.85558
3	0.570036
1	0.238775

6	0.819702
2	0.507189
8	0.750153
6	0.142722
7	0.134357
10	0.056601
10	0.295698
8	0.054062
3	0.597806
5	0.901795
14	0.287423
6	0.867584
3	0.232155
9	0.179734
3	0.481256
4	0.805266
10	0.744766
14	0.106221
9	0.76388
6	0.021238
6	0.668128
6	0.248307
1	0.384873
14	0.683542
3	0.976246
10	0.907971
14	0.808795
6	0.894404
5	0.486544
10	0.309157
8	0.442713
8	0.997306
14	0.665238
3	0.144932
3	0.300899
5	0.171661
14	0.901991
5	0.334745
14	0.324577
10	0.639173
10	0.49637
8	0.761162
7	0.716931
9	0.006479
10	0.350751
8	0.699574
5	0.111884
7	0.131815
7	0.137246
7	0.22912
6	0.734197
5	0.813131
8	0.030192
2	0.742353
8	0.710543
4	0.025444
4	0.387544
5	0.715987

2	0.253656
4	0.740039
10	0.022267
5	0.252559
7	0.508637
5	0.192267
6	0.323463
1	0.772145
10	0.918116
7	0.958832
9	0.642335
3	0.563756
2	0.75773
7	0.42914
3	0.17704
7	0.987004
3	0.24624
5	0.588797
14	0.979601
14	0.804418
2	0.069752
5	0.055007
9	0.935842
9	0.997668
10	0.056427
14	0.847039
1	0.66507
5	0.021039
1	0.736234
5	0.913417
14	0.271689
5	0.865076
6	0.305188
2	0.949054
14	0.766443
3	0.009159
1	0.173158
1	0.571219
10	0.058524
7	0.199181
14	0.267216
1	0.859792
8	0.146579
8	0.966488
2	0.48273
10	0.304254
6	0.821295
5	0.720632
9	0.7962
1	0.804341
2	0.103639
10	0.747454
1	0.238832
10	0.531472
6	0.572224
8	0.369456
8	0.241888
4	0.976973

9	0.090234
4	0.201318
9	0.342156
7	0.509263
6	0.299907
1	0.053641
8	0.699872
14	0.597394
8	0.655868
5	0.734563
1	0.21857
1	0.611843
5	0.323219
6	0.90449
5	0.962511
5	0.63429
3	0.55403
4	0.128131
10	0.536824
1	0.879272
7	0.901862
4	0.102561
8	0.139075
8	0.288787
14	0.757961
8	0.029566
8	0.633991
2	0.589766
14	0.863262
14	0.833915
1	0.301073
9	0.295468
14	0.86087
6	0.814221
14	0.085449
6	0.068925
5	0.557641
2	0.481714
9	0.441698
5	0.745887
2	0.81812
10	0.121234
2	0.311799
7	0.313139
8	0.253304
5	0.083203
14	0.17366
3	0.737778
9	0.568074
14	0.168046
7	0.372457
6	0.910159
9	0.786857
1	0.44367
6	0.531533
4	0.642567
2	0.138027
14	0.167535

9	0.2149
9	0.64388
1	0.704273
8	0.41555
6	0.885707
1	0.48385
7	0.569114
8	0.320532
14	0.315138
14	0.323615
4	0.93982
4	0.333921
14	0.991378
3	0.8263
3	0.963731
14	0.957002
14	0.415783
9	0.14355
9	0.553217
7	0.056075
5	0.363041
14	0.794472
4	0.597284
10	0.129318
9	0.451495
7	0.733023
9	0.989593
2	0.350029
7	0.5441
8	0.305401
3	0.876283
8	0.107571
9	0.785936
14	0.575316
6	0.827219
10	0.469922
4	0.209637
7	0.314167
6	0.312866
3	0.518971
6	0.265298
5	0.340011
5	0.688772
2	0.494406
9	0.728053
8	0.341486
7	0.120157
3	0.906635
3	0.699277
8	0.715825
5	0.929435
7	0.107409
14	0.44434
14	0.01901
1	0.312221
3	0.558841
2	0.480603
7	0.618599

14	0.305119
1	0.9011
7	0.669934
6	0.773414
7	0.879566
8	0.174146
6	0.361736
8	0.880792
10	0.625844
2	0.391254
6	0.516851
8	0.746008
14	0.472984
5	0.169739
14	0.717348
6	0.314241
8	0.547893
7	0.861953
5	0.330929
2	0.582593
6	0.104567
5	0.152422
4	0.525237
6	0.991574
3	0.999209
8	0.379874
9	0.17186
1	0.842143
5	0.943922
3	0.458046
14	0.093641
7	0.083174
3	0.370106
1	0.861279
8	0.964927
10	0.471669
8	0.360097
9	0.767115
3	0.858056
8	0.926163
9	0.242222
8	0.767104
6	0.733981
2	0.805322
14	0.972096
10	0.697624
5	0.963429
2	0.393588
5	0.914187
14	0.003129
2	0.265637
8	0.154971
9	0.423975
6	0.425631
9	0.340949
14	0.821606
9	0.287233
2	0.184727

4	0.815506
3	0.065509
7	0.959332
9	0.486396
3	0.738903
2	0.724991
6	0.973713
7	0.881695
10	0.283253
4	0.778478
9	0.399198
10	0.077337
2	0.12847
9	0.461347
6	0.694205
14	0.544619
3	0.942984
14	0.687344
5	0.233025
10	0.305242
8	0.534823
9	0.323858
8	0.342012
9	0.441912
3	0.190553
5	0.609432
1	0.954837
6	0.424486
2	0.809484
3	0.14325
5	0.749736
14	0.20653
3	0.009308
10	0.375091
6	0.56667
2	0.279245
9	0.932086
9	0.682131
8	0.507654
2	0.992886
2	0.901237
2	0.982567
2	0.214844
2	0.441228
4	0.901044
9	0.470881
10	0.882347
10	0.334414
14	0.804492
5	0.691126
3	0.174545
14	0.593132
4	0.153375
14	0.795
5	0.558234
8	0.090713
2	0.074656
1	0.958276

8	0.068011
4	0.971102
4	0.713159
8	0.492652
3	0.964153
9	0.383583
8	0.48997
1	0.514166
8	0.936106
7	0.905
14	0.294445
6	0.774986
1	0.442251
14	0.60449
5	0.148635
14	0.397578
3	0.446701
5	0.743438
4	0.593794
8	0.565283
14	0.505711
10	0.707283
14	0.691361
3	0.86605
8	0.343031
5	0.508957
7	0.06386
1	0.434685
9	0.210848
8	0.409167
8	0.266426
14	0.356498
6	0.153056
6	0.242266
4	0.422871
9	0.584578
6	0.429316
2	0.165885
1	0.237369
7	0.228264
5	0.942476
5	0.866716
6	0.358718
9	0.935457
8	0.386447
8	0.849176
14	0.442683
3	0.88309
7	0.553388
9	0.384594
7	0.884217
8	0.906875
9	0.968219
6	0.662518
2	0.348929
8	0.730662
14	0.103045
5	0.031967

3	0.420131
10	0.587811
8	0.766151
1	0.36313
9	0.41085
3	0.400016
14	0.857656
10	0.488013
8	0.170229
9	0.918414
3	0.067507
1	0.302653
7	0.211693
4	0.59276
9	0.971064
5	0.163984
4	0.449234
14	0.488892
10	0.008079
2	0.501413
1	0.378552
2	0.931554
14	0.792209
